# MRI T2 and T1ρ relaxation in patients at risk for knee osteoarthritis: a systematic review and meta-analysis

**DOI:** 10.1186/s12891-019-2547-7

**Published:** 2019-05-01

**Authors:** Hayden F. Atkinson, Trevor B. Birmingham, Rebecca F. Moyer, Daniel Yacoub, Lauren E. Kanko, Dianne M. Bryant, Jonathan D. Thiessen, R. Terry Thompson

**Affiliations:** 1 School of Physical Therapy, Faculty of Health Sciences, University of Western Ontario, London, Ontario Canada; 2 Wolf Orthopaedic Biomechanics Laboratory, Fowler Kennedy Sport Medicine Clinic, University of Western Ontario, London, Ontario Canada; 3 Bone and Joint Institute, University of Western Ontario, London, Ontario Canada; 4 School of Physiotherapy, Faculty of Health, Dalhousie University, Halifax, Nova Scotia Canada; 5 Faculty of Health Sciences, University of Western Ontario, London, Ontario Canada; 6 Schulich School of Medicine and Dentistry, University of Western Ontario, London, Ontario Canada; 7 Imaging Program, Lawson Health Research Institute, London, Ontario Canada; 8 Musculoskeletal Rehabilitation, Elborn College, University of Western Ontario, London, Ontario N6G 1H1 Canada

**Keywords:** T2 relaxation, T1ρ relaxation, Articular cartilage, Knee osteoarthritis, Imaging biomarker

## Abstract

**Background:**

Magnetic resonance imaging (MRI) T2 and T1ρ relaxation are increasingly being proposed as imaging biomarkers potentially capable of detecting biochemical changes in articular cartilage before structural changes are evident. We aimed to: 1) summarize MRI methods of published studies investigating T2 and T1ρ relaxation time in participants at risk for but without radiographic knee OA; and 2) compare T2 and T1ρ relaxation between participants at-risk for knee OA and healthy controls.

**Methods:**

We conducted a systematic review of studies reporting T2 and T1ρ relaxation data that included both participants at risk for knee OA and healthy controls. Participant characteristics, MRI methodology, and T1ρ and T2 relaxation data were extracted. Standardized mean differences (SMDs) were calculated within each study. Pooled effect sizes were then calculated for six commonly segmented knee compartments.

**Results:**

55 articles met eligibility criteria. There was considerable variability between scanners, coils, software, scanning protocols, pulse sequences, and post-processing. Moderate risk of bias due to lack of blinding was common. Pooled effect sizes indicated participants at risk for knee OA had lengthened T2 relaxation time in all compartments (SMDs from 0.33 to 0.74; *p* < 0.01) and lengthened T1ρ relaxation time in the femoral compartments (SMD from 0.35 to 0.40; *p* < 0.001).

**Conclusions:**

T2 and T1ρ relaxation distinguish participants at risk for knee OA from healthy controls. Greater standardization of MRI methods is both warranted and required for progress towards biomarker validation.

**Electronic supplementary material:**

The online version of this article (10.1186/s12891-019-2547-7) contains supplementary material, which is available to authorized users.

## Background

Magnetic resonance imaging (MRI) is commonly used to study knee osteoarthritis (OA), largely because of its ability to visually detect morphological changes in soft tissues [1–6]. However, in addition to visualizing structures within a joint, the measurable characteristics of MRI enable the quantification of tissue biochemistry, often termed compositional MRI.

Although several types of compositional MRI techniques exist, the vast majority of research in OA focuses on knee articular cartilage T2 and T1ρ relaxation times as these are suggested to show considerable promise and be clinically feasible [7–10]. Although the reported strengths of the correlations are variable, T2 and T1ρ relaxation times are associated with the composition of the extracellular matrix. T2 relaxation is inversely correlated with collagen network organization and structure, and is directly correlated with free water content [7]. Changes in T1ρ relaxation appear to be less specific, yet are also sensitive to changes in the extracellular matrix [8–14]. When the extracellular matrix of articular cartilage is compromised, characteristic of early biochemical processes in OA, water moves more freely within the cartilage, prolonging both MRI T2 and T1ρ relaxation time [13, 15, 16].

T2 and T1ρ relaxation have engendered considerable interest as a potential biomarkers for knee OA [17], especially given their proposed ability to detect biochemical changes in articular cartilage before structural changes are evident [15, 18, 19]. If these measures can detect compromised articular cartilage prior to radiographic evidence of OA, they may have the potential to serve as an outcome measure in early intervention studies targeting at-risk populations, such as people with knee anterior cruciate ligament (ACL) rupture [20–22], meniscal injuries [23, 24], or obesity [25, 26]. While this may be true of other compositional MRI measures (such as sodium, glycosaminoglycan chemical exchange saturation transfer [gagCEST], delayed gadolinium enhanced MRI of cartilage [dGEMRIC] [27]), T2 and T1ρ relaxation are perhaps the most clinically feasible, do not require a contrast agent, and are the focus of numerous studies that may enable meta-analysis when investigating their potential use as a biomarker.

Previous systematic reviews are encouraging in that they suggest T2 and T1ρ measures can be highly reliable when similar testing methods are used [27], and can distinguish between articular cartilage of healthy controls and patients with established radiographic OA [27, 28]. There are established criteria, however, for biomarker validation and qualification [29–31]. These include the ability to consistently measure the biomarker across testing sites [32, 33]. The extent to which previous studies investigating compositional MRI have used similar collection and analysis methods is presently unclear, and has been recently called into question [34]. Moreover, the potential utility of a biomarker to detect changes in the composition of knee articular cartilage relies on its ability to do so early in the disease process, before degenerative joint changes are evident on x-ray. Although there is abundant evidence suggesting T2 and T1ρ relaxation times are prolonged in knees with established radiographic OA compared to healthy knees [27, 28], the ability to detect changes between knees at risk for OA and healthy knees is less clear.

Therefore, purposes of this systematic review and meta-analysis were to: 1) summarize the MRI methods of published studies investigating T2 and T1ρ relaxation times in participants at risk for but without radiographic knee OA; and 2) compare T2 and T1ρ relaxation values between participants at-risk for knee OA and healthy controls.

## Methods

This systematic review follows the Preferred Reporting Items for Systematic Reviews and Meta-Analyses (PRISMA) guidelines [35] (Additional file 1: Appendix 5) (PROSPERO ID: CRD42018088352).

### Literature search

We sought the assistance of a research librarian to develop the search strategy. We searched the following electronic databases from their inception to June 2018: MEDLINE, EMBASE, Scopus, Cumulative Index to Nursing & Allied Health Literature (CINAHL), SPORTDiscus, and Web of Science, in addition to hand searching reference lists of included articles. Combined and truncated keywords and subject headings included “*magnetic resonance imaging* OR *compositional magnetic resonance imaging*” AND “*T2 mapping* OR *T1rho mapping* OR *T2 relaxation* OR *T1rho relaxation*” AND “*osteoarthritis* OR *articular cartilage*” AND “*knee* OR *tibiofemoral* OR *patellofemoral*”. A full example of the search strategy is provided in Additional file 1: Appendix 1.

### Eligibility criteria

Eligible studies included those published in English that reported T2 and/or T1ρ relaxation time in knee articular cartilage in at least two groups of participants including one group with any of the criteria commonly accepted for being at risk for knee OA, and a control group without any of those criteria. All study designs were considered. We used the Osteoarthritis Initiative (OAI) Incidence cohort criteria [36] to define a list of criteria for participants at risk for knee OA. These criteria include native knee symptoms in the past 12 months, overweight or obesity, history of knee injury which would cause difficulty walking for at least a week, history of knee surgery, family history of OA, lifestyle factors such as occupational risk (i.e. repetitive knee bending, squatting, lifting, etc.), age 70 years or older, and Kellgren & Lawrence (KL) radiographic grading of 0 or 1 [37]. Studies that included at-risk knees and contralateral healthy knees within the same participant were also included. We excluded patients with KL grade 2 or higher. For studies with multiple follow-up time points, only the baseline T2 and/or T1ρ relaxation data were used in our meta-analyses. Two reviewers independently assessed the eligibility of each article in two stages. Two reviewers independently assessed all titles and abstracts identified by the search. Articles meeting the inclusion criteria, according to at least one reviewer, were obtained as full-text manuscripts for further review. Articles meeting the inclusion criteria after full-text review were accepted in the review. Reviewers discussed any conflicts at all stages and a consensus was achieved.

### Data extraction

Two reviewers independently extracted T2 and T1ρ relaxation time of knee articular cartilage in six primary compartments: medial femoral condyle (MF), medial tibial plateau (MT), lateral femoral condyle (LF), lateral tibial plateau (LT), patellar cartilage (P), and trochlear groove of the femur (TrF) cartilage. If authors presented laminar differences (superficial and deep cartilage as separate regions of interest) the data from both regions were pooled. Given the variability in defining anterior, central, and posterior subregions of the femur and tibia across studies, we pooled the identified subregions (where necessary) to best analyze the load-bearing regions of the femoral condyles (generally in the region of the anterior horn of the meniscus to the posterior horn of the meniscus). For the P and TrF, we pooled all subregions (where necessary) to obtain a single value for the P or TrF. Reviewers discussed any conflicts and achieved consensus in all cases. Reviewers independently extracted relaxation time means and standard deviations (SD) for each participant group. The same reviewers also extracted the following information from each article: sample size, participant demographics, risk factors for OA, MRI hardware, pulse sequences, and parameters. Authors were contacted when sufficient data were not reported. If data were not provided or unclear, we contacted the original authors using provided e-mail addresses. In the case of no reply from the authors, we extracted data from figures when available. We used Covidence systematic review and meta-analysis software (www.covidence.org) to extract data.

### Quality assessment

Two reviewers independently evaluated the methodological quality of each study using the Risk of Bias in Nonrandomized Studies of Interventions (ROBINS-I) tool [38], consisting of seven items to assess the internal validity of each study (confounding, participant selection, intervention classification, deviation from intervention, missing data, outcome measurement, and outcome selection). Each item was evaluated as a low, moderate, serious, or critical risk of bias. Disagreements between reviewers were resolved by consensus after initial independent evaluation.

### Data analyses

We assessed agreement between reviewers using the kappa (κ) statistic. We compared compositional MRI data by calculating pooled estimates with 95% confidence intervals (95% CIs) for standardized mean differences (SMDs) using random-effects models. When calculating pooled effect sizes, we weighted all SMDs based on the sample size of the respective study. For both T2 and T1ρ relaxation time, the SMD was calculated using the difference between healthy controls and participants at risk for knee OA, divided by the pooled SD. If a study had multiple groups at risk for knee OA, only the group with the lowest risk was included in the calculation of the overall pooled effect size, based on reported measures of disease severity (KL Grade, International Cartilage Repair Society [ICRS] grade, Outerbridge Score, Whole Organ MRI Score [WORMS], etc.). All meta-analyses were performed using the Comprehensive Meta-Analysis software program (V3, Biostat; https://www.meta-analysis.com). We interpreted the magnitude of the SMD using Cohen’s *d* as small (< 0.2), moderate (0.2–0.8) and large (> 0.8) and positive values representing prolonged relaxation times in participants at risk for OA [39]. We assessed publication bias using the Egger’s Regression test [40], and if present, further analyses were planned to explore treatment effects adjusted for selective reporting [41]. We assessed the proportion of variability associated with heterogeneity using the I^2^ statistic and Q statistic [42]. We interpreted the size of I^2^ as low (25%), moderate (50%) or high (75%) heterogeneity [42].

### Sensitivity analyses

We repeated the primary analyses after excluding all but one study (with the greatest sample size) that included OAI participants to ensure we included data from the same knee only once. We also repeated the analyses after excluding studies that used both limbs from the same participant.

In the event of substantial heterogeneity, we planned three subgroup analyses. These groups included participants with a history of ACL injury (based on physical exam, imaging, or surgical confirmation), participants at risk for patellofemoral OA (based on the OAI Incidence cohort criteria) [36], and participants with articular cartilage injuries based on MR imaging, arthroscopic ICRS grades, or Outerbridge scores [43, 44].

## Results

### Study selection & article screening

**Fig. 1:**
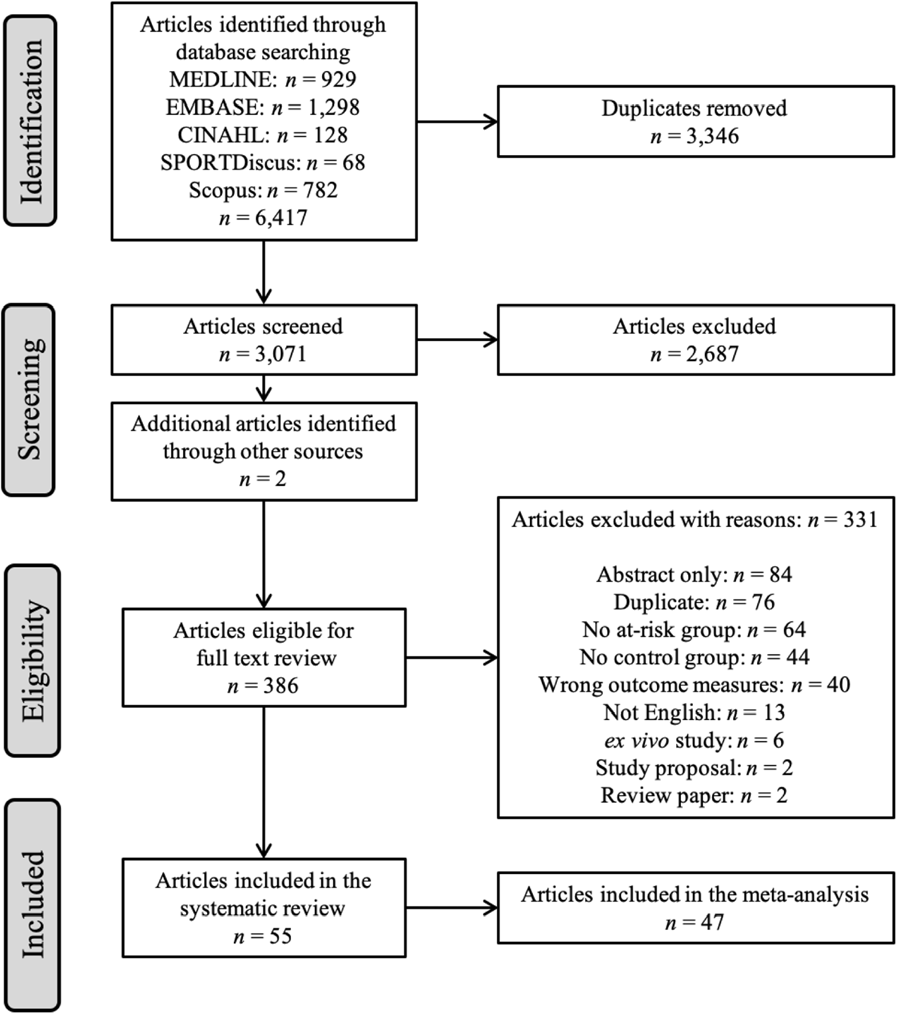
PRISMA flowchart quantifying studies accepted and rejected with reasons at different phases of review

We performed the initial search August 1st, 2018 and updated the search March 7th, 2019. We identified 6417 articles by the database search. After removing duplicates, we reviewed 3071 articles by title and abstract with excellent inter-rater agreement (κ =0.96) and 53 disagreements (1.7%) between reviewers. Disagreements were discussed, and after consensus, 386 articles were deemed eligible for full-text review (Fig. 1). After full text reviews, inter-rater agreement was excellent (κ =0.95), with 12 disagreements between reviewers. Disagreements were discussed, and after consensus, 55 articles met our inclusion criteria (Fig. 1) [15, 16, 20, 23, 24, 45–94], with a total of 3676 participants. Forty-seven studies were included in the meta-analysis, including data from 3079 participants. Articles included in the systematic review but excluded from the meta-analysis either examined incomparable regions of interest (ROI), or had insufficient data to be included in the meta-analyses [54, 66, 68, 69, 77, 85, 89, 90].

### Study characteristics

Characteristics of all studies included in the systematic review are described in Table 1 [15, 16, 20, 23, 24, 45–94]. T2 relaxation was included as an outcome measure in 38 studies, T1ρ relaxation was an outcome measure in 24 studies, and 8 of those studies evaluated both T2 and T1ρ relaxation. Studies varied considerably in terms of compositional MRI data acquisition and post-processing. Two different magnet strengths, four different manufacturers, 12 different magnet models, 16 different reported knee coils, 17 reported pulse sequences, and a wide variety of parameters were used to acquire compositional MRI data.

**Table 1:** Description of studies included in the systematic review

Authors	Participants	n (n_male_)	Age	Scanner	Coil	T1ρ Sequence (resolution)	TSL (ms)/SL Frequency (Hz)	T2 Sequence (resolution)	TR/TE
Amano et al. (2016)^c^	Control	19 (13)	31±5	3T GE	Invivo 8-Ch Tx/Rx	Sag 3D MAPSS (0.3×0.3×1.5)	0, 10, 40, 80/500	Sag 3D MAPSS (0.3×0.3×1.5)	4000/0, 13, 26, 51
	ACL injured	51 (29)	29±9						
van der Heijden et al. (2016)^c^	Control	70 (29)	23±6	3T GE	Invivo 8-Ch Tx/Rx	3D FSE (0.5×0.8×3.0) [3 slices/SR]	1, 16, 32, 64, 125/500	3D FSE (0.5×0.8×3.0) [3 slices/SR]	1263/3, 13, 27, 40, 68
	PFP	64 (35)	23±7						
Apprich et al. (2010)^bc^	ICRS Grade 0	14 (N/A)	37±8	3T Siemens	8-Ch Knee Array	--	--	Ax 2D MESE (0.4×0.4×3.0)	1200/14, 28, 41, 55, 69, 83
	ICRS Grade 1	5 (N/A)	37±8						
Apprich et al. (2012)^bc^	ICRS Grade 0	11 (N/A)	30±9	3T Siemens	8-Ch Knee Array	--	--	Ax 2D MESE (0.2×0.2×2.0)	1200/14, 28, 41, 55, 69, 83
	ICRS Grade 1	10 (N/A)	30±9						
Bae et al. (2015)^bc^	Uninjured Knee	10 (7)	34±8	3T Siemens	Siemens 8-Ch Tx/Rx	--	--	2D ME-FSE (0.5×0.5×3.0) [3 slices/SR]	1700/11, 21, 32, 42, 53, 64, 74, 85, 95, 106
	ACLR Knee	10 (7)	34±8						
Baum et al. (2012)^ac^	OAI Healthy	42 (21)	50±3	3T Siemens	Siemens 15-Ch Tx/Rx	--	--	Sag 2D MESE (0.3×0.4×3.0)	2700/10, 20, 30, 40, 50, 60, 70
	OAI Incidence	42 (21)	50±3						
Baum et al. (2013)^ac^	OAI Healthy	36 (11)	50±3	3T Siemens	Siemens 15-Ch Tx/Rx	--	--	Sag 2D MESE (0.3×0.4×3.0)	2700/10, 20, 30, 40, 50, 60, 70
	OAI Incidence	78 (33)	51±3						
Baum et al. (2012)^ac^	OAI Healthy	41 (15)	51±3	3T Siemens	Siemens 15-Ch Tx/Rx	--	--	Sag 2D MESE (0.3×0.4×3.0)	2700/10, 20, 30, 40, 50, 60, 70
	OAI Incidence	101 (50)	51±3						
Möstrom et al. (2015)^c^	Healthy Control	16 (9)	22±2	1.5T Philips	N/R	--	--	Sag 2D MESE (0.5×0.5×3.0)	2000/9, 18, 27, 36, 45, 54, 63, 72
	Patellar Dislocation	16 (9)	22±2						
Bining et al. (2009)^c^	Healthy Control	60 (N/A)	38±14	1.5T GE	Signa HD Tx/Rx	--	--	Sag 2D MESE (0.6×0.6×4.0)	1000/8, 16, 24, 32, 40, 48, 56, 64
	Cartilage Lesions	24 (N/A)	45±17						
Bolbos et al. (2008)^c^	Healthy Control	15 (11)	30±9	3T GE	Tx/Rx Quadrature Knee	Sag 3D SPGR (0.5×0.7×3.0)	0, 10, 40, 80/500	--	--
	ACL Rupture	16 (11)	33±6						
Farrokhi et al. (2011)^bc^	Healthy Control	10 (0)	27±4	3T GE	GE 8-Ch Knee	--	--	Sag 2D MESE (0.4×0.8×4.0) [3 slices/SR]	1800/20, 40, 60, 80
	PFP	10 (0)	28±4						
Gheno et al. (2016)^c^	Healthy Control	27 (22)	28±4	3T Philips	Invivo 8-Ch Tx/Rx	--	--	Sag 2D MESE (0.3×0.3×3.0)	3100/15, 30, 45, 60, 75, 90
	ACLR	27 (22)	29±5						
Van Ginckel et al. (2013)	Healthy Control	15 (8)	27±3	3T Siemens	8-Ch Knee	--	--	Sag 2D MESE (0.4×0.4×3.0)	1000/14 28, 41, 55, 69
	ACLR	15 (8)	27±1						
Gupta et al. (2014)^c^	Healthy Control	10 (8)	35±6	3T GE	Tx/Rx Quadrature Knee	Sag 3D SPGR (0.5×1.1×4.0) [3 slices/SR]	0, 10, 40, 80/500	--	--
	ACL Injury	10 (5)	39±6						
Haughom et al. (2012)^c^	Healthy Control	11 (4)	33±9	3T GE	8-Ch Tx/Rx	Sag 3D SPGR (0.3×0.6×1.5)	0, 10, 40, 80/500	--	--
	ACLR	11 (4)	33±9						
Hovis et al. (2011)^ac^	OAI Healthy	33 (8)	50±3	3T Siemens	N/R	--	--	Sag 2D MESE (0.3×0.4×3.0)	2700/10, 20, 30, 40, 50, 60, 70
	OAI Incidence	128 (57)	51±3						
Joseph et al. (2011)^ac^	OAI Healthy	53 (17)	50±3	3T Siemens	N/R	--	--	Sag 2D MESE (0.3×0.4×3.0)	2700/10, 20, 30, 40, 50, 60, 70
	OAI Incidence	93 (42)	51±3						
Kai et al. (2011) ^c^	Healthy Control	143 (72)	40±13	1.5T Siemens	Signa HD 8-Ch Tx/Rx	--	--	Sag 2D MESE (0.6×0.6×4.0)	1000/8, 16, 24, 32, 40, 48, 56, 64
	Meniscal Lesions	57 (27)	41±13						
Kang et al. (2016)^c^	Healthy Control	53 (15)	16±2	1.5T GE	N/R	--	--	Sag 2D MESE (0.5×0.9×4.0)	1500/9, 18, 27, 36, 45, 54, 63, 72, 81, 90, 99
	PF Instability	53 (15)	16±2						
Lansdown et al. (2015)^bc^	Healthy Control	10 (4)	31±5	3T GE	Invivo 8-Ch Tx/Rx	Sag 3D MAPSS (0.6×0.6×3.0)	0, 10, 40, 80/500	--	--
	ACLR	20 (8)	32±8						
Lau et al. (2016)^c^	Healthy Control	6 (3)	29	3T GE	Invivo 8-Ch Tx/Rx	2D FSE (0.3×0.6×1.5)	0, 10, 40, 80/500	--	--
	PFP	10 (2)	32						
Liebl et al. (2015)^a^	OAI Healthy	80 (30)	58±8	3T Siemens	USA Instruments Tx/Rx	--	--	Sag 2D MESE (0.3×0.4×3.0)	2700/10, 20, 30, 40, 50, 60, 70
	OAI Incidence	50 (22)	60±8						
H. Li et al. (2013)^c^	Healthy Control	15 (15)	27±5	3T Siemens	N/R	--	--	Sag 2D MESE (0.4×0.4×3.0) [5 slices/SR]	1523/14, 28, 41, 55, 69
	ACLR	30 (30)	29±5						
X. Li et al. (2011)^ac^	Healthy Control	10 (7)	34	3T GE	Quadrature Tx/Rx	Sag 3D SPGR (0.5×1.1×2.5)	20, 40. 60, 80/500	Sag 2D MESE (0.5×1.1×2.5)	2000/7, 12, 28, 60
	ACL Injured	12 (7)	34						
Matsubara et al. (2015)^c^	Healthy Control	19 (19)	39±7	3T Philips	8-Ch Tx/Rx	Sag 2D FFE (0.4×0.4×3.0) [3 slices/SR]	1, 20, 40, 60, 80/500	--	--
	Meniscal Tear	22 (15)	57±14						
Mosher et al. (2004)	18-30 y.o. Healthy	8 (0)	25±1	3T Bruker	Doty Scientific Litz	--	--	Sag 2D MESE (0.5×0.5×3.0)	1500/9, 18, 27, 36, 45, 54, 63, 72, 81, 90, 99, 108
	66-86 y.o. Healthy	7 (0)	75±7						
Okazaki et al. (2015)^c^	Healthy Control	6 (N/A)	32±2	3T Philips	8-Ch Tx/Rx	Sag 2D SPGR (0.4×0.4×3.0) [4 slices/SR]	1, 20, 40, 60, 80/500	--	--
	PCL Deficient	6 (N/A)	17±6						
Osaki et al. (2015)^c^	Healthy Control	14 (14)	37±6	3T Philips	8-Ch Tx/Rx	Sag 2D SPGR (0.4×0.4×3.0) [3 slices/SR]	1, 20, 40, 60, 80/500	--	--
	ACL Injured	49 (30)	25±9						
Palmieri-Smith et al. (2016)	Healthy Control	11 (5)	20±5	3T Philips	N/R	--	--	Sag 2D MESE (0.5×0.5×2.0)	1000/8, 16, 24, 32, 40, 48, 56, 64
	ACL Injured	11 (5)	19±6						
Pedoia et al. (2015)^c^	Healthy Control	15 (N/A)	32±5	3T GE	Invivo 8-Ch Tx/Rx	Sag 3D MAPSS (0.5×1.1×4.0)	0, 10, 40, 80/500	--	--
	ACL Injured	40 (N/A)	30±8						
Pedoia et al. (2016)^c^	Healthy Control	10 (5)	32±4	3T GE	Invivo 8-Ch Tx/Rx	Sag 3D MAPSS (0.5×1.1×4.0)	0, 10, 40, 80/500	--	--
	ACL Injured	52 (21)	28±12						
Pedoia et al. (2017)^c^	Healthy Control	15 (10)	31±5	3T GE	Invivo 8-Ch Tx/Rx	Sag 3D MAPSS (0.5×1.1×4.0)	0, 10, 40, 80/500	Sag 3D MAPSS (0.5×1.1×4.0)	4000/0, 14, 27, 55
	ACL Injured	40 (25)	30±8						
Rehnitz et al. (2014)^c^	Healthy Control	10 (N/A)	25	3T Siemens	Siemens 15-Ch Tx/Rx	--	--	Sag 2D MESE (0.4×0.4×3.0) [3 slices/SR]	1940/12, 24, 35, 47, 59, 71, 83, 94, 106, 118, 130, 142, 153
	Cartilage Lesions	40 (N/A)	47						
Russell et al. (2017)^c^	Healthy Control	15 (6)	57±9	3T GE	Invivo 8-Ch Tx/Rx	Sag 2D SPGR (0.5×1.1×4.0)	0, 2, 4, 6, 8, 12, 20, 40, 80/500	Sag 2D MESE (0.5×1.1×4.0)	4000/0, 2, 4, 7, 15, 29, 44, 58
	Cartilage Lesions	15 (6)	56±8						
Sauerschnig et al. (2014)^c^	Neutral	12 (4)	25±2	1.5T Siemens	Medical Advances 8-Ch	--	--	Sag 2D MESE (0.4×0.5×1.0)	1690/10, 20, 30, 40, 50, 60
	Varus Alignment	12 (10)	26±1						
	ACLR	40 (26)	35±8						
Snoj et al. (2016)^bc^	Healthy Controls	20 (11)	33±7	3T Siemens	Invivo 8-Ch Tx/Rx	--	--	Sag 2D MESE (0.4×0.4×3.0) [2 slices/SR]	1000/14, 28, 41, 55, 69, 83
	ACLR	40 (26)	35±8						
Subhawong et al. (2014)^c^	Healthy Control	28 (17)	31±10	3T Siemens	N/R	--	--	Sag 2D MESE (N/A) [1 slice/SR]	1650/13, 26, 39, 52, 65, 77
	PFP	22 (8)	34±13						
Su et al. (2013)^c^	Healthy Control	16 (8)	33	3T GE	Clinical MR Solutions Tx/Rx Quadrature	Cor 3D SPGR (0.5×0.7×4.0)	0, 10, 40, 80/500	Sag 3D SPGR (0.5×0.7×4.0)	2000/4, 15, 25, 46
	ACL Injured	15 (7)	35						
Su et al. (2016)^c^	Healthy Control	54 (31)	30±8	3T GE	Clinical MR Solutions Tx/Rx Quadrature	Cor 3D SPGR (0.5×0.7×4.0)	0, 10, 40, 80/500	Sag 3D SPGR (0.5×0.7×4.0)	2000/4, 15, 25, 46
	ACL Injured	54 31(31)	30±8						
Theologis et al. (2014)^bc^	Healthy Control	18 (8)	38±8	3T GE	Clinical MR Solutions Tx/Rx Quadrature	Sag 3D SPGR (0.5×0.7×4.0)	0, 10, 40, 80/500	--	--
	ACLR	18 (8)	38±8						
Thuiller et al. (2013)^c^	Healthy Control	10 (4)	31±3	3T GE	Invivo 8-Ch Tx/Rx	2D FSE (0.3×0.6×1.5)	0, 10, 40, 80/500	--	--
	PFP	20 (10)	31±5						
Wirth et al. (2016)^ac^	Healthy Control	89 (36)	55±8	3T Siemens	Siemens 15-Ch Tx/Rx	--	--	Sag 2D MESE (0.3×0.3×3.0)	2700/10, 20, 30, 40, 50, 60, 70
	Risk for OA	28 (14)	61±9						
Witschey et al. (2010)	Healthy Controls	9 (2)	N/R	1.5T Siemens	Invivo 8-Ch Tx/Rx	3D GRE (0.5×0.6×0.5)	--	--	--
	Cartilage Lesions	6 (3)	N/R						
Xu et al. (2011)	Healthy Controls	30 (18)	25	3T Philips	SENSE 8-Ch	--	--	Sag 2D MESE (0.5×0.3×3.0)	2400/15, 30, 45, 60, 75, 90
	Cartilage Injury	42 (25)	37						
Zaid et al. (2015)^bc^	Healthy Controls	25 (12)	28±7	3T GE	Invivo 8-Ch Tx/Rx	Sag 3D SPGR (0.5×1.1×4.0)	--	--	--
	ACLR	25 (12)	28±7						
Kim et al. (2018)^bc^	Healthy Control	10 (7)	34±8	3T Siemens	Siemens 8-Ch	--	--	Sag 2D MESE (0.5×0.5×3.0) [2 slices/SR]	1700/11, 21, 32, 42, 53, 64, 72, 85, 95, 106
	ACLR	10 (7)	34±8						
Kogan et al. (2018)^c^	Healthy Control	15 (10)	33±11	3T GE	NeoCoil 16-Ch Tx/Rx Flex	--	--	3D DESS (0.5×0.5×1.5)	24.6/5.8, 43.4
	ACL Injured	15 (10)	33±11						
Mostrom et al. (2017)^c^	Healthy Control	17 (5)	25±3	1.5T Philips	N/R	--	--	Sag 2D MESE (0.5×0.5×3.0)	2000/9, 18, 27, 36, 45, 54, 63, 72
	PF Instability	17 (5)	25±3						
Pfeiffer et al. (2017)^abc^	Healthy Control	21 (11)	24±3	3T Siemens	Siemens XR 80/200 Gradient Coil	3D FLASH (1.8×0.9×3.0)	0, 10, 20, 30, 40/500	--	--
	ACLR	21 (11)	24±3						
Pietrosimone et al. (2017)^bc^	Healthy Control	18 (8)	22±4	3T Siemens	Siemens 4-Ch Flex Coil	3D FLASH (0.6×1.3×3.0)	0, 10, 20, 30, 40/500	--	--
	ACLR	18 (8)	22±4						
Tao et al. (2018)^c^	Healthy Control	23 (7)	29±8	3T Siemens	Siemens 8-Ch Tx/Rx	--	--	Sag 2D MESE (0.4×0.4×3.0)	1523/13.8, 27.6, 41.4, 55.2, 69.0
	ACL Rupture	23 (5)	32±10						
Teng et al. (2017)^c^	Healthy Control	12 (8)	32±6	3T GE	Invivo 8-Ch Tx/Rx	Sag 3D MAPSS (0.5×1.1×4.0)	0, 10, 40, 80/500	Sag 3D MAPSS (0.5×1.1×4.0)	0, 14, 27, 55
	ACL Rupture	33 (20)	31±9						
Wang et al. (2018)^c^	Healthy Control	9 (4)	26±5	3T Siemens	Siemens 8-Ch	--	--	Sag 2D MESE (0.4×0.4×3.0) [3/5/10 slices/SR]	1200/14, 28, 41, 55, 69
	ACLR	28 (17)	30±6						
Collins et al. (2018)^b^	Normal BMI	8 (5)	30	3T Siemens	Invivo 8-Ch Tx/Rx	Sag 3D FISP (1.1×0.5×3.0)	3500/5, 10, 40, 80/500	--	--
	Obese BMI	7 (3)	32						

*ms* milliseconds, *SL* spin lock, *Hz* Hertz, *TR* Repetition time, *TE* echo time, *ACL* anterior cruciate ligament, *ACLR* ACL reconstruction, *GE* General Electric, *MAPSS* magnetization-prepared spoiled gradient echo, *SR* subregion, *PF* patellofemoral, *PFP* patellofemoral pain, *FFE* fast field echo, *FSE* fast spin echo, *ICRS* International Cartilage Rating Scale, *Ch* channel, *Sag* sagittal, *Cor* coronal, *Ax* axial, *MESE* multi-echo spin echo, *OAI* Osteoarthritis Initiative, *Tx* transmit, *Rx* receive, *N/R* not reported, *SPGR* spoiled gradient recalled echo, *FISP* fast imaging with steady state precession

^a^multicenter study

^b^use of pre-scan unloading protocol

^c^indicates post-processing methods that could be used in any dataset

### Quality assessment

Agreement between reviewers for all seven items in the ROBINS-I tool was moderate (κ =0.54, 95% CI = 0.48–0.61), with disagreements being primarily on the subjective severity of bias rather than the presence or absence of bias. Forty-five studies presented with a moderate overall risk of bias, seven presented with a serious risk of bias, and three presented with a low risk of bias. The most common sources of risk for bias was lack of blinding, or reporting of blinding, of the outcome assessors, as well as risk of bias in participant selection. No studies were excluded based on quality assessment. Results of the quality assessment are included in Additional file 1: Appendix 2.

### Descriptive analyses

Forty-seven out of 55 studies observed a significant increase in compositional MRI values in one or more regions of interest in the at-risk group compared to the healthy control group. Specifically, 31 of 38 studies assessing T2 relaxation time reported significant lengthening in the at-risk group, and 21 of 24 studies assessing T1ρ relaxation time reported significant lengthening in the at-risk group.

### Meta-analyses

**Fig. 2:**
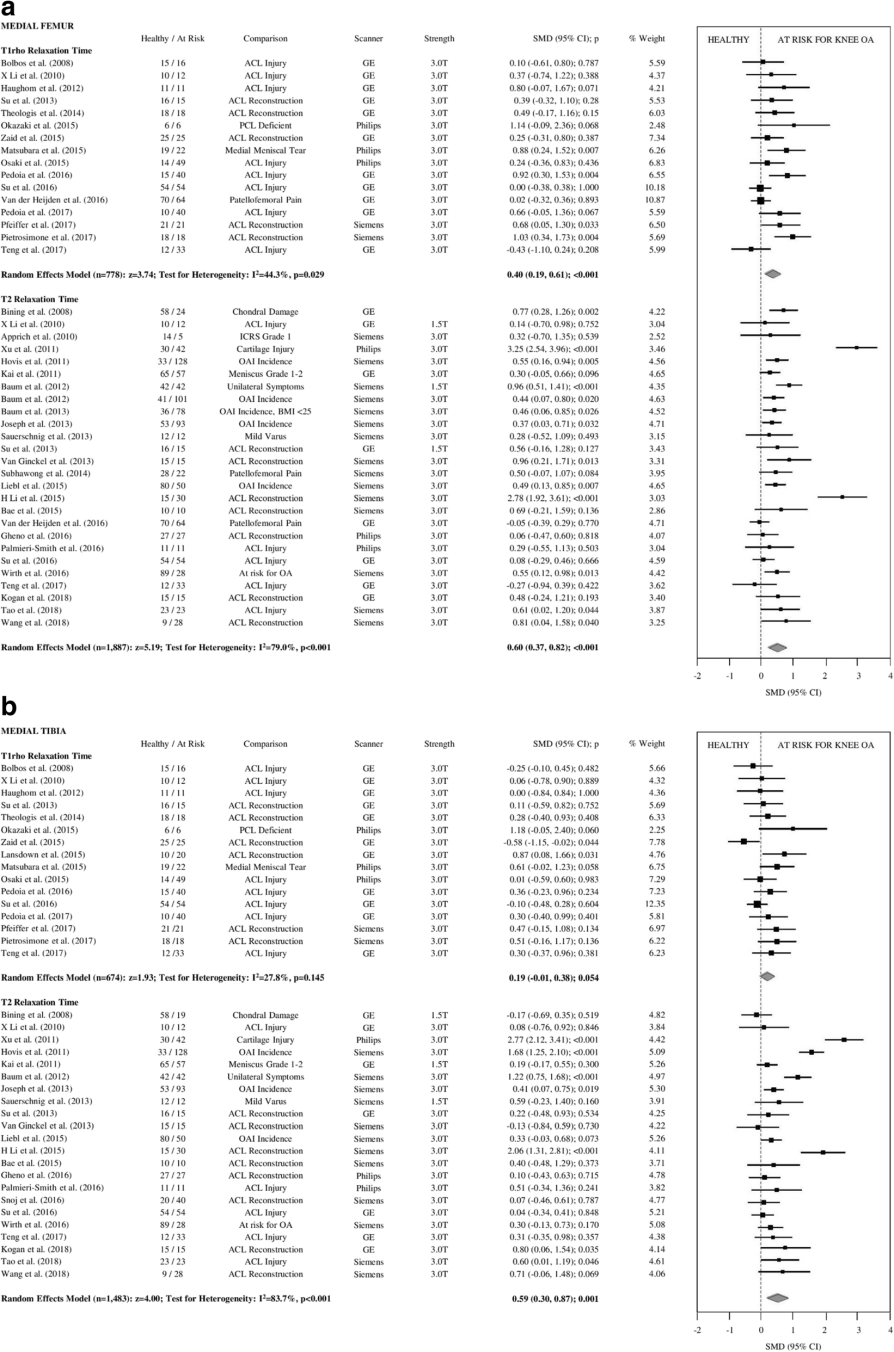
**a** Forest plots illustrating individual and pooled SMD for differences in T1rho and T2 relaxation time of medial femoral articular cartilage between healthy controls and participants at risk for knee OA. SMD = standardized mean difference, 95% CI = 95% confidence interval, ACL = anterior cruciate ligament, PCL = posterior cruciate ligament, ICRS=International Cartilage Repair Society, OAI=Osteoarthritis Initiative, OA = osteoarthritis, GE = General Electric, T = Tesla. **b** Forest plots illustrating individual and pooled SMD for differences in T1rho and T2 relaxation time of medial tibial articular cartilage between healthy controls and participants at risk for knee OA. SMD = standardized mean difference, 95% CI = 95% confidence interval, ACL = anterior cruciate ligament, PCL = posterior cruciate ligament, ICRS=International Cartilage Repair Society, OAI=Osteoarthritis Initiative, OA = osteoarthritis, GE = General Electric, T = Tesla

**Fig. 3:**
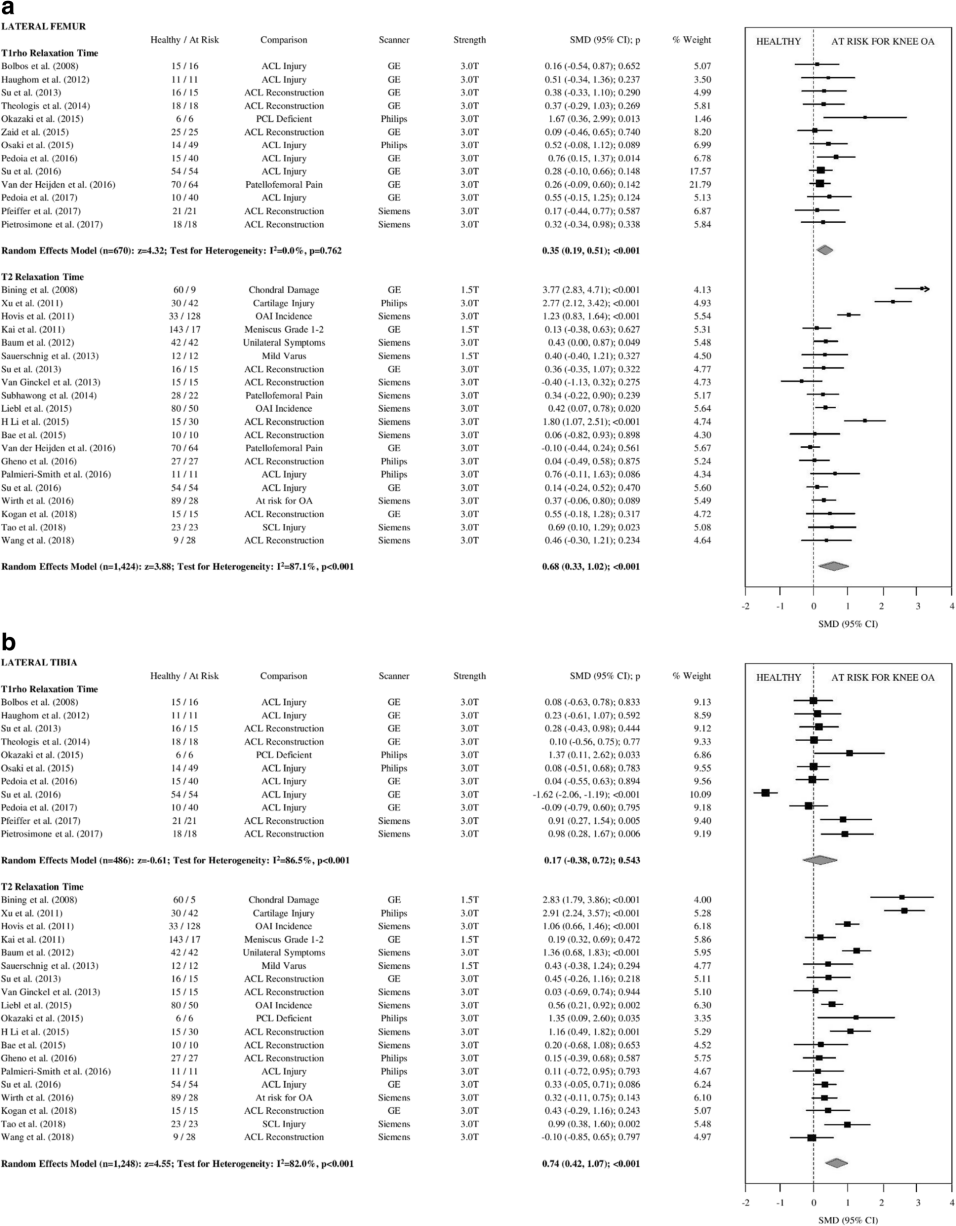
**a** Forest plots illustrating individual and pooled SMD for differences in T1rho and T2 relaxation time of lateral femoral articular cartilage between healthy controls and participants at risk for knee OA. SMD = standardized mean difference, 95% CI = 95% confidence interval, ACL = anterior cruciate ligament, PCL = posterior cruciate ligament, ICRS=International Cartilage Repair Society, OAI=Osteoarthritis Initiative, OA = osteoarthritis, GE = General Electric, T = Tesla. **b** Forest plots illustrating individual and pooled SMD for differences in T1rho and T2 relaxation time of lateral tibial articular cartilage between healthy controls and participants at risk for knee OA. SMD = standardized mean difference, 95% CI = 95% confidence interval, ACL = anterior cruciate ligament, PCL = posterior cruciate ligament, ICRS=International Cartilage Repair Society, OAI=Osteoarthritis Initiative, OA = osteoarthritis, GE = General Electric, T = Tesla

**Fig. 4:**
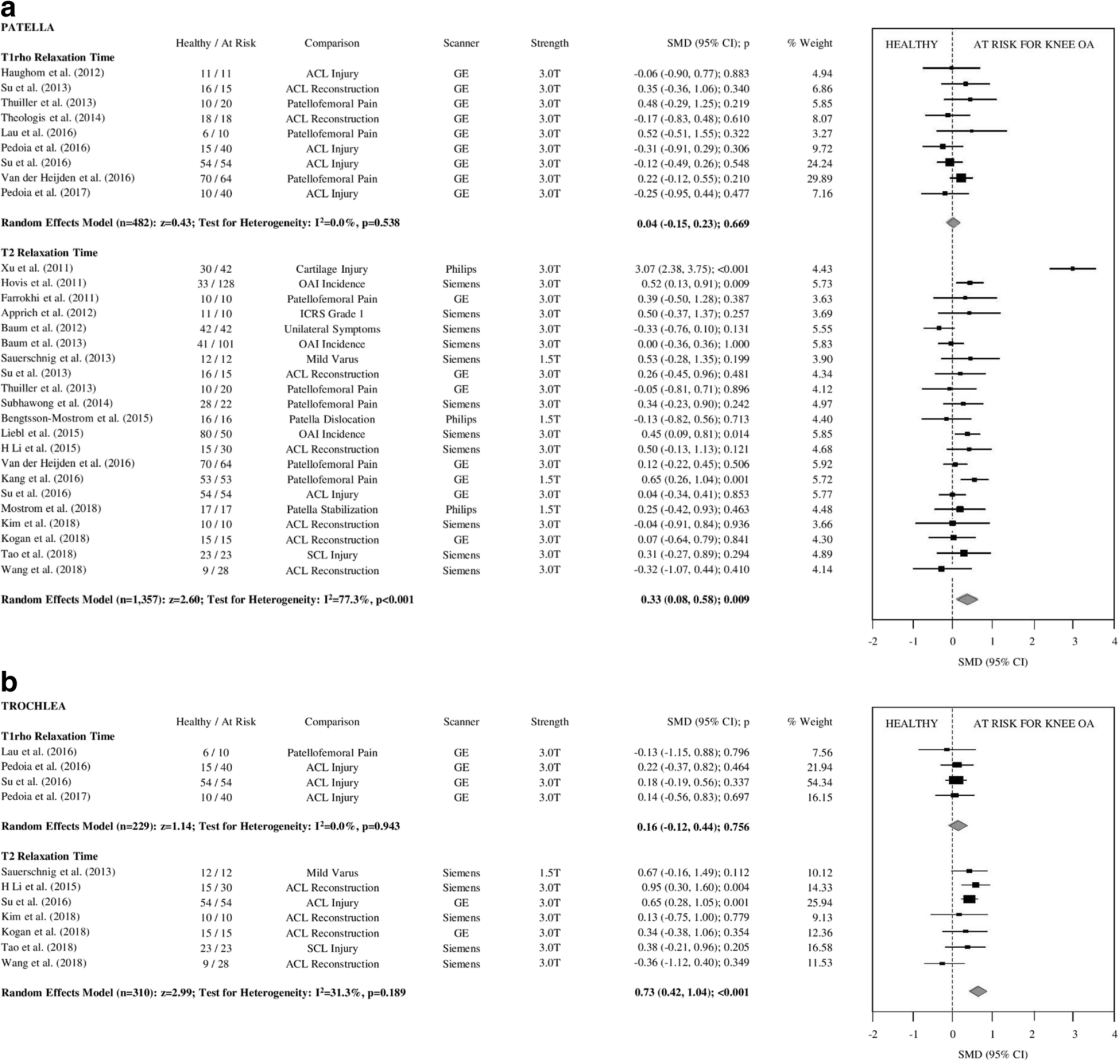
**a** Forest plots illustrating individual and pooled SMD for differences in T1rho and T2 relaxation time of patellar articular cartilage between healthy controls and participants at risk for knee OA. SMD = standardized mean difference, 95% CI = 95% confidence interval, ACL = anterior cruciate ligament, ICRS=International Cartilage Repair Society, OAI=Osteoarthritis Initiative, OA = osteoarthritis, GE = General Electric, T = Tesla. **b** Forest plots illustrating individual and pooled SMD for differences in T1rho and T2 relaxation time of trochlear articular cartilage between healthy controls and participants at risk for knee OA. SMD = standardized mean difference, 95% CI = 95% confidence interval, ACL = anterior cruciate ligament, ICRS=International Cartilage Repair Society, OAI=Osteoarthritis Initiative, OA = osteoarthritis, GE = General Electric, T = Tesla

We were able to pool data for T2 and/or T1ρ relaxation time for cartilage ROIs in the MF and LF, MT and LT, P, and TrF cartilage. Forest plots, including individual and pooled SMDs are presented in Figs. 2, 3, and 4.

At-risk knees had significantly prolonged T2 relaxation times for all compartments, small-to-moderate effect sizes (SMD = 0.33–0.74; *p* < 0.001; Figs. 2, 3, and 4). At-risk knees had significantly prolonged T1ρ relaxation times for the MF and LF with small effect sizes (SMD = 0.35–0.40; *p* < 0.001; Figs. 2a, 3a). There were no significant differences in T1ρ relaxation between groups for the MT, LT, P, or TrF compartments (SMD = 0.04–0.19, *p* > 0.05–0.76; Figs. 2b, 3b and 4b).

### Publication Bias and heterogeneity

Egger’s regression test for publication bias was not significant for any meta-analysis assessing pooled SMD of T2 relaxation time. For T1ρ relaxation time, meta-analyses of the MF and LT compartments showed significant evidence of publication bias (*p* < 0.01). After using Duval & Tweedie’s trim and fill method [41] to correct for publication bias, T1ρ relaxation time of the MF was not significantly different in participants at risk for knee OA (SMD = 0.16[95% CI:-0.07;0.40]; *p* = 0.17). After adjustment for publication bias, T1ρ relaxation time of the LT remained non-significant (SMD = 0.17[95% CI:-0.38;0.71]; *p* = 0.54).

For meta-analyses assessing T2 relaxation time, heterogeneity was significant for all analyzed compartments (I^2^ = 77–87%; *p* < 0.01) except for the TrF compartment (I^2^ = 31%; *p* = 0.19). Four studies consistently contributed to the heterogeneity of T2 relaxation SMD, including two studies fitting in the cartilage injury subgroup. Removal of these studies resulted in non-significant heterogeneity in the MF and P compartments (I^2^ = 19–23%; *p* > 0.2); however, heterogeneity remained high after removal of outliers in the MT and LF compartments (I^2^ = 66–70%, *p* > 0.01). After removal of outliers, T2 relaxation time remained significantly prolonged for those at risk for knee OA. For meta-analyses assessing T1ρ relaxation time, heterogeneity was significant for the MF and LT compartments (I^2^ = 44–87%; *p* < 0.01), and non-significant for all other compartments (I^2^ = 0–28%; *p* = 0.15–0.94). The trim & fill method [41] is limited in its ability to identify publication bias in heterogeneous datasets where no true bias exists [95]. Thus there may be no significant publication bias for the heterogeneous SMD’s of T1ρ in the MF and LT compartments.

### Sensitivity analyses

We performed two sensitivity analyses. The first analysis excluded all but one study (6 articles excluded) using OAI data to ensure no subjects in the meta-analyses were used more than once. Effect sizes remained moderate and significant in all compartments (SMD = 0.38–0.73; *p* < 0.02). The second analysis excluded all studies which used within-patient comparisons (healthy knee versus at-risk knee). Following exclusion of articles (6 articles for T2, 6 for T1ρ), effect sizes remained moderate-to-large for T2 (all compartments: SMD = 0.42–0.83; *p* < 0.1), and remained moderate for some T1ρ compartments (MF, MT, LF: SMD = 0.27–0.37; *p* < 0.02) and remained small and non-significant for others (LT, P, TrF: SMD = 0.13–0.14; *p* > 0.29. Detailed results of sensitivity analyses can be found in Additional file 1: Appendix 3.

### Subgroup analyses

We performed three subgroup analyses to determine respective effect sizes for patients with ACL injury, risk for patellofemoral OA, and articular cartilage lesions. Results of the subgroup analyses suggested that SMDs controls were small-to-moderate for the ACL-injury subgroup compared to controls (14 articles for T2: SMD = 0.13–0.56; *p* = 0.002–0.27. 14 articles for T1ρ: SMD = -0.11–0.30; *p* = 0.001–0.8). We obtained similar small-to-moderate effect sizes for the patellofemoral OA risk subgroup (8 articles for T2: SMD = 0.06–0.20; *p* = 0.004–0.78. 3 articles for T1ρ: SMD = -0.13–0.28; *p* = 0.06–0.89). These effect sizes were generally smaller compared to the remainder of the sample in the primary analysis. The articular cartilage injury subgroup demonstrated large effect sizes (4 articles for T2: SMD = 1.29–2.88; p = 0.001–0.38) which were larger in comparison to the remainder of the sample in the primary analysis. Detailed results of subgroups analyses can be found in Additional file 1: Appendix 4.

## Discussion

The present pooled within-study effect sizes that combine data from 47 studies involving 3661 participants suggest T2 and T1ρ relaxation times distinguish between healthy participants and participants at risk for knee OA. The present results are consistent with the only other published systematic review we are aware of [23], yet extends its findings by focusing on persons at-risk for but without radiographic knee OA, and by providing a thorough summary of the variable T2 and T1ρ collection, processing, and analysis methods. Strengths of the present study include adherence to well-established guidelines for conducting systematic reviews and meta-analyses [35]. These include multiple reviewers reaching consensus at each step of the literature search, study selection and data extraction; assessment of study quality; assessment and adjustment for publication bias; and pre-planned meta-analyses including sensitivity analyses based on a priori hypotheses in the event of substantial heterogeneity. Limitations of the present meta-analyses may include pooling participants at risk, as there are likely several different phenotypes for the development of OA [90]. Our subgroup analyses suggest that T2 and T1ρ values of articular cartilage are slightly different across participants with various risk factors, and future research should explore those differences further. A common methodological limitation in the studies included in this review is the lack of blinding and/or reporting of blinding procedures. Other limitations include those inherent to cross-sectional versus prospective designs that measure change in patient status over time.

Importantly, there was considerable variability between MRI methods, including scanners, coils, software, scanning protocols, pulse sequences, and post-processing, which can all influence T2 or T1ρ relaxation. For example, knee articular cartilage T2 relaxation time is inversely proportional to magnetic field strength [96], and can differ significantly when using different brands of scanners of the same advertised field strength [97]. In this review alone, four brands of scanners, and two magnet strengths were identified across studies (Table 1). T2 relaxation time is significantly prolonged when using a phased-array knee coil compared to a quadrature transmit receive knee coil [98]. Sixteen different knee coils were used in studies in this review (Table 1), with a wide variety of phased-array and quadrature coils. Choice of pulse sequence can also significantly affect relaxation time, with a difference of as much as 10 ms observed across commonly used sequences [99, 100]. Knowledge of the context and collection methods is important when comparing compositional MRI values across the literature, as a 1.8 ms increase in T2 relaxation time is representative of a 1% increase in free water content when comparing within the same participant [101, 102]. Seventeen different pulse sequences were used to collect the data presented in this review (Table 1). Pre-scan unloading protocol is an important consideration that varies across studies, as T2 relaxation time increases with unloading time due to water reuptake into the cartilage [93]. Post-processing and segmentation can also affect T2 and T1ρ values, such as how the assessor defines the ROI, ROI variance between studies, number of slices included in the ROI [103], proximity of borders to other tissues, and partial volume effects [104]. Continued use of proposed standardized nomenclature and ROI definition will improve comparability of ROI’s across studies and sites [105]. Taken together, these findings identify substantial differences in methods across testing sites, suggest considerable caution should be adopted when making comparisons across studies, and highlight the limitation in the current state of T2 or T1ρ relaxation as imaging biomarkers.

These findings suggest future use of compositional MRI measures as potential biomarkers would benefit considerably from a greater understanding of the effects of different testing methods [106] and greater standardization of data collection and analysis measures [34]. The importance of greater standardization across testing sites is underscored by the variability in results of studies evaluating the test-retest reliability of compositional MRI measures, even when the exact same methods are used [28]. For example, studies evaluating test-retest reliability using the same testing conditions report intra-class correlation coefficients (ICC) ranging from 0 to 0.98 [107, 108], and coefficients of variation (CV) ranging from 1.7 to 22.2 [65, 96–98, 106, 109–116]. Fewer studies evaluating test-retest reliability using similar methods but different scanner manufacturers suggest ICCs ranging from 0.2 to 0.93 [107], and CVs ranging from 2.3 to 6.3 [97, 106]. Arguably, the most important consideration regarding improved reliability of compositional MRI as an imaging biomarker is comparability of values across scanners and centers. The present findings therefore support current international efforts from researchers and vendors to improve sequences, calibration, and standardization [17], such as the Radiological Society of North America Quantitative Imaging Biomarker Alliance [117], and multicenter studies such as the OAI [118]. In addition to these efforts, another approach may be the use of calibration phantoms [119] to develop correction functions to account for varying hardware and software used by different centers [17].

By pooling within-study comparisons, the present primary analysis indicates that T2 and T1ρ relaxation times in articular cartilage are significantly prolonged in knees at risk for developing OA, especially in the more commonly affected compartments. T2 relaxation time was significantly prolonged in participants at risk for knee OA in all analyzed compartments with effect sizes ranging from small-to-moderate (SMD = 0.33–0.74; *p* < 0.001), suggesting T2 is sensitive to early changes in collagen orientation and structural integrity [120], as well as water content in these at-risk participants [13, 15, 16]. These findings add support to the use of T2 relaxation time for early detection of OA, before substantial radiographic changes are evident, and support further efforts towards compositional MRI biomarker validation and qualification.

Interestingly, effect sizes for T1ρ relaxation time were small, and lower for each analyzed compartment in comparison to effect sizes for T2 relaxation time, (SMD = 0.04–0.40; *p* = 0.001–0.76), and only the MF and LF compartments demonstrated significantly prolonged T1ρ relaxation time compared to healthy controls (SMD = 0.35–0.40; *p* < 0.001). However, there were fewer studies that included T1ρ as an outcome measure with generally smaller sample sizes. More research comparing T2 and T1ρ relaxation times for participants at various stages of knee OA is required.

In all knee compartments, there was significant heterogeneity associated with the overall pooled effect sizes for T2 relaxation time (Figs. 2, 3, and 4). Sensitivity analysis suggested that the high effect sizes of the cartilage injury subgroups are responsible for this heterogeneity (SMD = 1.29–2.88; p = 0.001–0.38), and after removal from the analyses, heterogeneity was no longer significant in the MF and P compartments (I^2^ = 19–23%; *p* > 0.2) but remained moderate in the MT and LF compartments (I^2^ = 66–70, *p* > 0.01). There were no articles assessing T1ρ relaxation time of participants with cartilage injury, which may explain the lack of heterogeneity in the T1ρ meta-analyses. The large effect sizes observed in these studies including patients with cartilage injury may be due to the different mechanopathology as a result of focal defects [18] in comparison to other participants in this systematic review. Alternatively, we must acknowledge the substantial difference in age between this at-risk subgroup and controls. Publication bias was also significant in three compartments for T1ρ relaxation time, which may be due to the relative novelty of such measures in comparison to T2 relaxation time. There was no publication bias observed in any meta-analyses assessing T2 relaxation time.

## Conclusions

Based on these results, T2 and T1ρ relaxometry of articular cartilage show substantial promise in their ability to identity pathological cartilage in participants at risk for knee OA. The present results are consistent with cross-sectional studies reporting known risk factors, such as increased age [89], body mass [42], and knee malalignment [111], and their association with significantly prolonged articular cartilage T2 relaxation times. The present study also highlights the wide variety of methods currently used to collect, process, and analyze T2 and T1ρ mapping. Overall, the present results emphasize both the potential, as well as the need for greater standardization of methods across sites for T2 and T1ρ data collection and processing procedures to make greater gains toward potential biomarker validation.

## Additional file

Additional file 1: Appendix 1 Search Strategy List of terms used to search the databases for eligible studies in the systematic review and meta-analyses. Appendix 2 Title of Data: Risk of Bias in Non-randomized Studies - of Interventions (ROBINS-I) Summary of the quality assessment for all studies using the ROBINS-I tool, grading studies on seven domains (confounding, participant selection bias, intervention bias, deviation from intervention, missing data, outcome measurement bias, outcome reporting bias) and their associated risk of bias (low, moderate, or severe). Appendix 3 Summary of Sensitivity Analyses Results of sensitivity analyses to account for potential bias of duplicate inclusions of participants as part of the Osteoarthritis Initiative, as well as potential for bias of studies using within-subject designs (healthy knee versus at-risk knee within the same participant). Appendix 4 Summary of Subgroup Analyses Results of subgroup analyses to investigate potential differences in effect sizes for groups with specific risk factors (anterior cruciate ligament injury, risk for patellofemoral osteoarthritis, and articular cartilage injuries. Appendix 5 Preferred Reporting of Items in Systematic Reviews and Meta-Analyses (PRISMA) Checklist PRISMA table identifying where in the text all required aspects of the checklist can be found in the manuscript. (DOCX 40 kb)

## Abbreviations

95% CI: 95% confidence interval
ACL: Anterior cruciate ligament
CINAHL: Cumulative Index to Nursing & Allied Health Literature
CV: Coefficient of variation
dGEMRIC: Delayed gadolinium enhanced magnetic resonance imaging of cartilage
gagCEST: Glycosaminoglycan chemical exchange saturation transfer
ICC: Intra-class correlation coefficient
ICRS: International Cartilage Repair Society
KL: Kellgren & Lawrence
LF: Lateral femur
LT: Lateral tibia
MF: Medial femur
MRI: Magnetic resonance imaging
MT: Medial tibia
OA: Osteoarthritis
OAI: Osteoarthritis Initiative
P: Patella
PRISMA: Preferred Reporting Items for Systematic Reviews and Meta-Analyses
ROBINS-I: Risk of Bias in Non-randomized Studies - of Interventions
ROI: Region of interest
SD: Standard deviation
SMD: Standardized mean difference
TrF: Trochlea of femur

## References

[1] Eckstein F, Cicuttini F, Raynauld J-P, Waterton JC, Peterfy C (2006). Magnetic resonance imaging (MRI) of articular cartilage in knee osteoarthritis (OA): morphological assessment. Osteoarthr Cartil.

[2] Amin S, LaValley M, Guermazi A (2005). The relationship between cartilage loss on magnetic resonance imaging and radiographic progression in men and women with knee osteoarthritis. Arthritis Rheum.

[3] Conaghan PG, Felson D, Gold G, Lohmander S, Totterman S, Altman R (2006). MRI and non-cartilaginous structures in knee osteoarthritis. Osteoarthr Cartil.

[4] Roemer FW, Guermazi A, Felson DT, et al. Presence of MRI-detected joint effusion and synovitis increases the risk of cartilage loss in knees without osteoarthritis at 30-month follow-up: the MOST study. Ann Rheum Dis. 2011. 10.1136/ard.2011.150243. Accessed 10 Sept 2018. PMC3496084 21791448

[5] Felson DT (2011). Imaging abnormalities that correlate with joint pain. Br J Sports Med.

[6] Terzidis IP, Christodoulou AG, Ploumis AL, Metsovitis SR, Koimtzis M, Givissis P (2004). The appearance of kissing contusion in the acutely injured knee in the athletes. Br J Sports Med.

[7] Nieminen MT, Rieppo J, Töyräs J (2001). *T*_2_ relaxation reveals spatial collagen architecture in articular cartilage: a comparative quantitative MRI and polarized light microscopic study. Magn Reson Med.

[8] Keenan KE, Besier TF, Pauly JM (2011). Prediction of glycosaminoglycan content in human cartilage by age, T1ρ and T2 MRI. Osteoarthr Cartil.

[9] Duvvuri U, Kudchodkar S, Reddy R, Leigh JS (2002). T1ρ relaxation can assess longitudinal proteoglycan loss from articular cartilage in vitro. Osteoarthr Cartil.

[10] van Tiel J, Kotek G, Reijman M (2016). Is T1ρ mapping an alternative to delayed gadolinium-enhanced MR imaging of cartilage in the assessment of Sulphated glycosaminoglycan content in human osteoarthritic knees? An in Vivo Validation Study. Radiology.

[11] Mlynárik V, Trattnig S, Huber M, Zembsch A, Imhof H (1999). The role of relaxation times in monitoring proteoglycan depletion in articular cartilage. J Magn Reson Imaging.

[12] Mlynárik V, Szomolányi P, Toffanin R, Vittur F, Trattnig S (2004). Transverse relaxation mechanisms in articular cartilage. J Magn Reson.

[13] Menezes N, Gray ML, Hartke JR, Deborah B (2004). T2 and T1ρ MRI in articular cartilage systems. Magn Reson Med.

[14] Taylor C, Carballido-Gamio J, Majumdar S, Li X (2009). Comparison of quantitative imaging of cartilage for osteoarthritis: T2, T1ρ, dGEMRIC and contrast-enhanced computed tomography. Magn Reson Imaging.

[15] Liebl H, Joseph G, Nevitt MC (2015). Early T2 changes predict onset of radiographic knee osteoarthritis: data from the osteoarthritis initiative. Ann Rheum Dis.

[16] Mosher TJ, Dardzinski BJ (2004). Cartilage MRI T2 relaxation time mapping: overview and applications. Seminars in Musculoskeletal Radiology.

[17] Link TM, Li X (2018). Establishing compositional MRI of cartilage as a biomarker for clinical practice. Osteoarthr Cartil.

[18] Baum T, Joseph GB, Karampinos DC, Jungmann PM, Link TM, Bauer JS (2013). Cartilage and meniscal T2 relaxation time as non-invasive biomarker for knee osteoarthritis and cartilage repair procedures. Osteoarthr Cartil.

[19] Prasad AP, Nardo L, Schooler J, Joseph GB, Link TM (2013). T1ρ and T2 relaxation times predict progression of knee osteoarthritis. Osteoarthr Cartil.

[20] Li X, Kuo D, Theologis A (2011). Cartilage in anterior cruciate ligament–reconstructed knees: MR imaging T1 _ρ_ and T2—initial experience with 1-year follow-up. Radiology..

[21] Su F, Pedoia V, Teng H-L (2016). The association between MR T1ρ and T2 of cartilage and patient-reported outcomes after ACL injury and reconstruction. Osteoarthr Cartil.

[22] Klocke Noelle F., Amendola Annunziato, Thedens Daniel R., Williams Glenn N., Luty Christopher M., Martin James A., Pedersen Douglas R. (2013). Comparison of T1ρ, dGEMRIC, and Quantitative T2 MRI in Preoperative ACL Rupture Patients. Academic Radiology.

[23] Kai B, Mann SA, King C, Forster BB (2011). Integrity of articular cartilage on T2 mapping associated with meniscal signal change. Eur J Radiol.

[24] Matsubara H, Okazaki K, Takayama Y (2015). Detection of early cartilage deterioration associated with meniscal tear using T1ρ mapping magnetic resonance imaging. BMC Musculoskelet Disord.

[25] Jungmann PM, Kraus MS, Alizai H (2013). Association of Metabolic Risk Factors with Cartilage Degradation Assessed by T2 relaxation time at the knee: data from the osteoarthritis initiative. Arthritis Care Res (Hoboken)..

[26] Serebrakian AT, Poulos T, Liebl H (2015). Weight loss over 48 months is associated with reduced progression of cartilage T2 relaxation time values: data from the osteoarthritis initiative. J Magn Reson Imaging.

[27] MacKay J.W., Low S.B.L., Smith T.O., Toms A.P., McCaskie A.W., Gilbert F.J. (2018). Systematic review and meta-analysis of the reliability and discriminative validity of cartilage compositional MRI in knee osteoarthritis. Osteoarthritis and Cartilage.

[28] Atkinson HF, Birmingham T, Moyer R, Kanko L, Yacoub D, Giffin J (2017). T1rho and T2 relaxation of knee articular cartilage in patients with and at risk for knee osteoarthritis: a systematic review and meta-analysis. Osteoarthr Cartil.

[29] Bauer DC, Hunter DJ, Abramson SB (2006). Review classification of osteoarthritis biomarkers: a proposed approach. Osteoarthr Cartil.

[30] European Society of Radiology (ESR) ES of R (2010). White paper on imaging biomarkers. Insights Imaging.

[31] Abramson RG, Burton KR, J-PJ Y (2015). Methods and challenges in quantitative imaging biomarker development. Acad Radiol.

[32] Group F-NBW. BEST (Biomarkers, EndpointS, and Other Tools) Resource. Food and Drug Administration (US); 2016. https://www.ncbi.nlm.nih.gov/books/NBK326791/. Accessed 28 Aug 2018. 27010052

[33] Hunter DJ, Nevitt M, Losina E, Kraus V (2014). Biomarkers for osteoarthritis: current position and steps towards further validation. Best Pract Res Clin Rheumatol.

[34] Roemer FW, Kijowski R, Guermazi A (2017). Editorial: from theory to practice – the challenges of compositional MRI in osteoarthritis research. Osteoarthr Cartil.

[35] Moher D, Liberati A, Tetzlaff J, Altman DG, Group TP (2009). Preferred reporting items for systematic reviews and meta-analyses: the PRISMA statement. PLoS Med.

[36] Nevitt MC, Felson DT, Lester G. OAI protocol the osteoarthritis initiative protocol for the cohort study. Osteoarthr Initiat. 2006:1–74 https://oai.epi-ucsf.org/datarelease/docs/StudyDesignProtocol.pdf. Accessed 18, July 2018.

[37] Kellgren J. H., Lawrence J. S. (1957). Radiological Assessment of Osteo-Arthrosis. Annals of the Rheumatic Diseases.

[38] Sterne JA, Hernán MA, Reeves BC (2016). ROBINS-I: a tool for assessing risk of bias in non-randomised studies of interventions. BMJ..

[39] Cohen J. Statistical power analysis for the behavioral sciences second edition. 10.1016/C2013-0-10517-X.

[40] Rothstein HR, Sutton AJ, Borenstein M. Publication Bias in Meta-analysis. 2005. 10.1002/0470870168.index.

[41] Duval S, Tweedie R (2000). Trim and fill: a simple funnel-plot-based method of testing and adjusting for publication Bias in meta-analysis. Biometrics..

[42] Higgins JPT, Thompson SG, Deeks JJ, Altman DG (2003). Measuring inconsistency in meta-analyses. BMJ..

[43] van den Borne MPJ, Raijmakers NJH, Vanlauwe J (2007). International cartilage repair society (ICRS) and Oswestry macroscopic cartilage evaluation scores validated for use in autologous chondrocyte implantation (ACI) and microfracture. Osteoarthr Cartil.

[44] Outerbridge R. E. (1961). THE ETIOLOGY OF CHONDROMALACIA PATELLAE. The Journal of Bone and Joint Surgery. British volume.

[45] Baum T, Joseph GB, Arulanandan A (2012). Association of magnetic resonance imaging-based knee cartilage T2 measurements and focal knee lesions with knee pain: data from the osteoarthritis initiative. Arthritis Care Res (Hoboken).

[46] Baum T, Joseph GB, Nardo L (2013). Correlation of magnetic resonance imaging-based knee cartilage T2 measurements and focal knee lesions with body mass index: thirty-six-month followup data from a longitudinal, observational multicenter study. Arthritis Care Res (Hoboken)..

[47] Baum T, Stehling C, Joseph GB (2012). Changes in knee cartilage T2 values over 24 months in subjects with and without risk factors for knee osteoarthritis and their association with focal knee lesions at baseline: data from the osteoarthritis initiative. J Magn Reson Imaging.

[48] Bengtsson Moström E, Lammentausta E, Finnbogason T, Weidenhielm L, Janarv P-M, Tiderius CJ (2015). Pre- and postcontrast T1 and T2 mapping of patellar cartilage in young adults with recurrent patellar dislocation. Magn Reson Med.

[49] Bining HJS, Santos R, Andrews G, Forster BB (2009). Can T2 relaxation values and color maps be used to detect chondral damage utilizing subchondral bone marrow edema as a marker?. Skelet Radiol.

[50] Bolbos RI, Ma CB, Link TM, Majumdar S, Li X (2008). In vivo T1rho quantitative assessment of knee cartilage after anterior cruciate ligament injury using 3 tesla magnetic resonance imaging. Investig Radiol.

[51] Farrokhi S, Colletti PM, Powers CM (2011). Differences in patellar cartilage thickness, transverse relaxation time, and deformational behavior. Am J Sports Med.

[52] Gheno R, Yoon YC, Wang JH, Kim K, Baek S-Y (2016). Changes in the *T*_2_ relaxation value of the tibiofemoral articular cartilage about 6 months after anterior cruciate ligament reconstruction using the double-bundle technique. Br J Radiol.

[53] Van Ginckel A, Verdonk P, Victor J, Witvrouw E (2013). Cartilage status in relation to return to sports after anterior cruciate ligament reconstruction. Am J Sports Med.

[54] Gupta R, Virayavanich W, Kuo D (2014). MR T1ρ quantification of cartilage focal lesions in acutely injured knees: correlation with arthroscopic evaluation. Magn Reson Imaging.

[55] Haughom B, Schairer W, Souza RB, Carpenter D, Ma CB, Li X (2012). Abnormal tibiofemoral kinematics following ACL reconstruction are associated with early cartilage matrix degeneration measured by MRI T1rho. Knee..

[56] Hovis KK, Stehling C, Souza RB (2011). Physical activity is associated with magnetic resonance imaging-based knee cartilage T2 measurements in asymptomatic subjects with and those without osteoarthritis risk factors. Arthritis Rheum.

[57] Joseph GB, Baum T, Carballido-Gamio J (2011). Texture analysis of cartilage T2 maps: individuals with risk factors for OA have higher and more heterogeneous knee cartilage MR T2 compared to normal controls - data from the osteoarthritis initiative. Arthritis Res Ther..

[58] Kang CH, Kim HK, Shiraj S, Anton C, Kim DH, Horn PS (2016). Patellofemoral instability in children: T2 relaxation times of the patellar cartilage in patients with and without patellofemoral instability and correlation with morphological grading of cartilage damage. Pediatr Radiol.

[59] Lansdown DA, Allen C, Zaid M (2015). A comprehensive in vivo kinematic, quantitative MRI and functional evaluation following ACL reconstruction — a comparison between mini-two incision and anteromedial portal femoral tunnel drilling. Knee..

[60] Lau BC, Thuillier DU, Pedoia V (2016). Inter- and intra-rater reliability of patellofemoral kinematic and contact area quantification by fast spin echo MRI and correlation with cartilage health by quantitative T1ρ MRI. Knee..

[61] Li H, Tao H, Hua Y, Chen J, Li Y, Chen S (2013). Quantitative magnetic resonance imaging assessment of cartilage status: a comparison between young men with and without anterior cruciate ligament reconstruction. Arthrosc J Arthrosc Relat Surg..

[62] Okazaki K, Takayama Y, Osaki K (2015). Subclinical cartilage degeneration in young athletes with posterior cruciate ligament injuries detected with T1ρ magnetic resonance imaging mapping. Knee Surgery, Sport Traumatol Arthrosc..

[63] Osaki K, Okazaki K, Takayama Y (2015). Characterization of biochemical cartilage change after anterior cruciate ligament injury using T1ρ mapping magnetic resonance imaging. Orthop J Sport Med.

[64] Palmieri-Smith RM, Wojtys EM, Potter HG (2016). Early cartilage changes after anterior cruciate ligament injury: evaluation with imaging and serum biomarkers—a pilot study. Arthrosc J Arthrosc Relat Surg.

[65] Pedoia V, Li X, Su F, Calixto N, Majumdar S (2016). Fully automatic analysis of the knee articular cartilage *T*_1ρ_ relaxation time using voxel-based relaxometry. J Magn Reson Imaging.

[66] Pedoia V, Russell C, Randolph A, Li X, Majumdar S, AF-ACL Consortium A-A (2016). Principal component analysis-T1ρ voxel based relaxometry of the articular cartilage: a comparison of biochemical patterns in osteoarthritis and anterior cruciate ligament subjects. Quant Imaging Med Surg.

[67] Pedoia V, Su F, Amano K (2017). Analysis of the articular cartilage T _1ρ_ and T _2_ relaxation times changes after ACL reconstruction in injured and contralateral knees and relationships with bone shape. J Orthop Res.

[68] Rehnitz C, Kupfer J, Streich NA (2014). Comparison of biochemical cartilage imaging techniques at 3 T MRI. Osteoarthr Cartil.

[69] Russell C, Pedoia V, Souza RB, Majumdar S (2017). Cross-sectional and longitudinal study of the impact of posterior meniscus horn lesions on adjacent cartilage composition, patient-reported outcomes and gait biomechanics in subjects without radiographic osteoarthritis. Osteoarthr Cartil.

[70] Sauerschnig M, Bauer JS, Kohn L (2014). Alignment does not influence cartilage T2 in asymptomatic knee joints. Knee Surgery, Sport Traumatol Arthrosc..

[71] Snoj Ž, Zupanc O, Salapura V (2016). Retrospective quantitative cartilage and semi-quantitative morphological evaluation at 6 years after ACL reconstruction. Arch Orthop Trauma Surg.

[72] Subhawong TK, Thakkar RS, Padua A, Flammang A, Chhabra A, Carrino JA (2014). Patellofemoral friction syndrome: magnetic resonance imaging correlation of morphologic and T2 cartilage imaging. J Comput Assist Tomogr.

[73] Su F, Hilton JF, Nardo L (2013). Cartilage morphology and T1ρ and T2 quantification in ACL-reconstructed knees: a 2-year follow-up. Osteoarthr Cartil.

[74] Theologis AA, Haughom B, Liang F (2014). Comparison of T1rho relaxation times between ACL-reconstructed knees and contralateral uninjured knees. Knee Surgery, Sport Traumatol Arthrosc.

[75] Thuillier DU, Souza RB, Wu S, Luke A, Li X, Feeley BT (2013). T _1ρ_ imaging demonstrates early changes in the lateral Patella in patients with patellofemoral pain and Maltracking. Am J Sports Med.

[76] Wirth W, Maschek S, Roemer FW, Eckstein F (2016). Layer-specific femorotibial cartilage T2 relaxation time in knees with and without early knee osteoarthritis: data from the osteoarthritis initiative (OAI). Sci Rep.

[77] Witschey WRT, Borthakur A, Fenty M (2010). T1ρ MRI quantification of arthroscopically confirmed cartilage degeneration. Magn Reson Med.

[78] Xu J, Xie G, Di Y, Bai M, Zhao X (2011). Value of T2-mapping and DWI in the diagnosis of early knee cartilage injury. J Radiol Case Rep.

[79] Zaid M, Lansdown D, Su F (2015). Abnormal tibial position is correlated to early degenerative changes one year following ACL reconstruction. J Orthop Res.

[80] Kim C-W, Hosseini A, Lin L (2018). Quantitative analysis of T2 relaxation times of the patellofemoral joint cartilage 3 years after anterior cruciate ligament reconstruction.

[81] Kogan F, Fan AP, Monu U, Iagaru A, Hargreaves BA, Gold GE (2018). Quantitative imaging of bone-cartilage interactions in ACL-injured patients with PET-MRI. Osteoarthr Cartil.

[82] Moström EB, Lammentausta E, Finnbogason T, Weidenhielm L, Janarv P-M, Tiderius CJ (2017). T2 mapping and post-contrast T1 (dGEMRIC) of the patellar cartilage: 12-year follow-up after patellar stabilizing surgery in childhood. Acta Radiol Open.

[83] Pfeiffer S, Harkey MS, Stanley LE (2018). Associations between slower walking speed and T1ρ magnetic resonance imaging of femoral cartilage following anterior cruciate ligament reconstruction. Arthritis Care Res (Hoboken)..

[84] Pietrosimone B, Nissman D, Padua DA (2017). Associations between cartilage proteoglycan density and patient outcomes 12 months following anterior cruciate ligament reconstruction. Knee..

[85] Amano K, Pedoia V, Su F, Souza RB, Li X, Ma CB (2016). Persistent biomechanical alterations after ACL reconstruction are associated with early cartilage matrix changes detected by quantitative MR. Orthop J Sport Med..

[86] Tao Hongyue, Qiao Yang, Hu Yiwen, Xie Yuxue, Lu Rong, Yan Xu, Chen Shuang (2018). Quantitative T2-Mapping and T2⁎-Mapping Evaluation of Changes in Cartilage Matrix after Acute Anterior Cruciate Ligament Rupture and the Correlation between the Results of Both Methods. BioMed Research International.

[87] Teng H-L, Wu D, Su F (2017). Gait characteristics associated with a greater increase in medial knee cartilage T1rho and T2 relaxation times in patients undergoing anterior cruciate ligament reconstruction. Am J Med.

[88] Wang X, Wrigley TV, Bennell KL (2018). Cartilage quantitative T2 relaxation time 2-4 years following isolated anterior cruciate ligament reconstruction. J Orthop Res.

[89] Mosher TJ, Liu Y, Yang QX (2004). Age dependency of cartilage magnetic resonance imaging T2 relaxation times in asymptomatic women. Arthritis Rheum.

[90] Collins AT, Kulvaranon ML, Cutcliffe HC (2018). Obesity alters the in vivo mechanical response and biochemical properties of cartilage as measured by MRI. Arthritis Res Ther.

[91] van der Heijden RA, Oei EHG, Bron EE (2016). No difference on quantitative magnetic resonance imaging in patellofemoral cartilage composition between patients with patellofemoral pain and healthy controls. Am J Sports Med.

[92] Apprich S, Welsch GH, Mamisch TC (2010). Detection of degenerative cartilage disease: comparison of high-resolution morphological MR and quantitative T2 mapping at 3.0 tesla. Osteoarthr Cartil.

[93] Apprich S, Mamisch TC, Welsch GH (2012). Quantitative T2 mapping of the patella at 3.0 T is sensitive to early cartilage degeneration, but also to loading of the knee. Eur J Radiol.

[94] Klocke Noelle F., Amendola Annunziato, Thedens Daniel R., Williams Glenn N., Luty Christopher M., Martin James A., Pedersen Douglas R. (2013). Comparison of T1ρ, dGEMRIC, and Quantitative T2 MRI in Preoperative ACL Rupture Patients. Academic Radiology.

[95] Terrin N, Schmid CH, Lau J, Olkin I (2003). Adjusting for publication bias in the presence of heterogeneity. Stat Med.

[96] Welsch GH, Apprich S, Zbyn S (2011). Biochemical (T2, T2* and magnetisation transfer ratio) MRI of knee cartilage: feasibility at ultra-high field (7T) compared with high field (3T) strength. Eur Radiol.

[97] Balamoody S, Williams TG, Wolstenholme C (2013). Magnetic resonance transverse relaxation time T2 of knee cartilage in osteoarthritis at 3-T: a cross-sectional multicentre, multivendor reproducibility study. Skelet Radiol.

[98] Dardzinski BJ, Schneider E (2013). Radiofrequency (RF) coil impacts the value and reproducibility of cartilage spin–spin (T2) relaxation time measurements. Osteoarthr Cartil.

[99] Matzat SJ, McWalter EJ, Kogan F, Chen W, Gold GE (2015). T _2_ relaxation time quantitation differs between pulse sequences in articular cartilage. J Magn Reson Imaging.

[100] Pai A, Li X, Majumdar S (2008). A comparative study at 3 T of sequence dependence of T2 quantitation in the knee. Magn Reson Imaging.

[101] Lüsse S, Knauss R, Werner A, Gründer W, Arnold K (1995). Action of compression and cations on the proton and deuterium relaxation in cartilage. Magn Reson Med.

[102] Lüssea S, Claassen H, Gehrke T (2000). Evaluation of water content by spatially resolved transverse relaxation times of human articular cartilage. Magn Reson Imaging.

[103] Crawley AP, Henkelman RM (1987). Errors inT2 estimation using multislice multiple-echo imaging. Magn Reson Med.

[104] Eckstein F, Heudorfer L, Faber SC, Burgkart R, Englmeier K-H, Reiser M (2002). Long-term and resegmentation precision of quantitative cartilage MR imaging (qMRI). Osteoarthr Cartil.

[105] Eckstein F, Ateshian G, Burgkart R (2006). Proposal for a nomenclature for magnetic resonance imaging based measures of articular cartilage in osteoarthritis. Osteoarthr Cartil.

[106] Li X, Pedoia V, Kumar D (2015). Cartilage T1ρ and T2 relaxation times: longitudinal reproducibility and variations using different coils, MR systems and sites. Osteoarthr Cartil.

[107] Mosher TJ, Zhang Z, Reddy R (2011). Knee articular cartilage damage in osteoarthritis: analysis of MR image biomarker reproducibility in ACRIN-PA 4001 multicenter trial. Radiology..

[108] Singh A, Haris M, Cai K, Kogan F, Hariharan H, Reddy R (2014). High resolution T1ρ mapping of in vivo human knee cartilage at 7T. Zadpoor AA, ed. PLoS One.

[109] Li X, Han ET, Ma CB, Link TM, Newitt DC, Majumdar S (2005). In vivo 3T spiral imaging based multi-slice T1ρ mapping of knee cartilage in osteoarthritis. Magn Reson Med.

[110] Liess C, Lüsse S, Karger N, Heller M, Glüer C-C (2002). Detection of changes in cartilage water content using MRI T2-mapping in vivo. Osteoarthr Cartil.

[111] Liu F, Choi KW, Samsonov A (2015). Articular cartilage of the human knee joint: in vivo multicomponent T2 analysis at 3.0 T. Radiology..

[112] Carballido-Gamio J, Link TM, Majumdar S (2008). New techniques for cartilage magnetic resonance imaging relaxation time analysis: texture analysis of flattened cartilage and localized intra- and inter-subject comparisons. Magn Reson Med.

[113] Duryea J, Cheng C, Schaefer LF, Smith S, Madore B (2016). Integration of accelerated MRI and post-processing software: a promising method for studies of knee osteoarthritis. Osteoarthr Cartil.

[114] Hannila I, Susanna Räinä S, Tervonen O, Ojala R, Nieminen MT (2009). Topographical variation of T2 relaxation time in the young adult knee cartilage at 1.5 T. Osteoarthr Cartil.

[115] Jordan CD, McWalter EJ, Monu UD (2014). Variability of CubeQuant T1ρ, quantitative DESS T2, and cones sodium MRI in knee cartilage. Osteoarthr Cartil.

[116] Li X, Wyatt C, Rivoire J (2014). Simultaneous acquisition of T _1ρ_ and T _2_ quantification in knee cartilage: repeatability and diurnal variation. J Magn Reson Imaging.

[117] Buckler AJ, Bresolin L, Dunnick NR, Sullivan DC (2011). Group F the. A collaborative Enterprise for Multi-Stakeholder Participation in the advancement of quantitative imaging. Radiology..

[118] Peterfy CG, Schneider E, Nevitt M (2008). The osteoarthritis initiative: report on the design rationale for the magnetic resonance imaging protocol for the knee. Osteoarthr Cartil.

[119] Keenan KE, Ainslie M, Barker AJ (2018). Quantitative magnetic resonance imaging phantoms: a review and the need for a system phantom. Magn Reson Med.

[120] Xia Y, Moody JB, Burton-Wurster N, Lust G (2001). Quantitative in situ correlation between microscopic MRI and polarized light microscopy studies of articular cartilage. Osteoarthr Cartil.

