# Supplementary material for: MRI T2 and T1ρ relaxation in patients at risk for knee osteoarthritis: a systematic review and meta-analysis
Source: BMC Musculoskelet Disord. 2019 May 1;20:182. doi: 10.1186/s12891-019-2547-7 (PMC6492327; doi:10.1186/s12891-019-2547-7)
Supplement: Supplementary file 1 — Appendix 1 Search Strategy List of terms used to search the databases for eligible studies in the systematic review and meta-analyses. Appendix 2 Title of Data: Risk of Bias in Non-randomized Studies - of Interventions (ROBINS-I) Summary of the quality assessment for all studies using the ROBINS-I tool, grading studies on seven domains (confounding, participant selection bias, intervention bias, deviation from intervention, missing data, outcome measurement bias, outcome reporting bias) and their associated risk of bias (low, moderate, or severe). Appendix 3 Summary of Sensitivity Analyses Results of sensitivity analyses to account for potential bias of duplicate inclusions of participants as part of the Osteoarthritis Initiative, as well as potential for bias of studies using within-subject designs (healthy knee versus at-risk knee within the same participant). Appendix 4 Summary of Subgroup Analyses Results of subgroup analyses to investigate potential differences in effect sizes for groups with specific risk factors (anterior cruciate ligament injury, risk for patellofemoral osteoarthritis, and articular cartilage injuries. Appendix 5 Preferred Reporting of Items in Systematic Reviews and Meta-Analyses (PRISMA) Checklist PRISMA table identifying where in the text all required aspects of the checklist can be found in the manuscript. (DOCX 40 kb) [file 12891_2019_2547_MOESM1_ESM.docx]

**APPENDICES**

Appendix 1. The following is an example of the full search strategy used for MEDLINE.

1. magnetic resonance imaging.mp. or exp Magnetic Resonance Imaging/
2. magnetic resonance.mp.
3. MRI.mp.
4. compositional magnetic resonance imaging.mp.
5. compositional MRI.mp
6. qMRI.mp.
7. quantitative magnetic resonance imaging.mp
8. 1 or 2 or 3 or 4 or 5 or 6 or 7
9. T1p.mp. or T1rho.mp
10. T1p relaxation.mp. or T1rho relaxation.mp
11. T1 mapping.mp. or T1rho mapping.mp
12. T1p mapping.mp.
13. T2.mp.
14. T2 relaxation.mp.
15. T2 mapping.mp.
16. relaxation time.mp.
17. 9 or 10 or 11 or 12 or 13 or 14 or 15 or 16
18. osteoarthritis.mp. or exp Osteoarthritis/ or exp Osteoarthritis, Knee/
19. articular cartilage.mp. or exp Cartilage, Articular/
20. hyaline cartilage.mp. or Hyaline Cartilage/ or Cartilage/
21. proteoglycan.mp. or exp Proteoglycans/
22. glycosaminoglycan.mp. or Glycosaminoglycans/
23. exp Collagen/ or collagen.mp.
24. 18 or 19 or 20 or 21 or 22 or 23
25. Knee/ or Knee Joint/ or knee.mp.
26. tibiofemoral.mp.
27. Patellofemoral Joint/ or patellofemoral.mp.
28. 25 or 26 or 27
29. 8 and 17 and 24 and 28

Appendix 2. Results of the quality assessment using the Risk of Bias in Nonrandomized Studies of Interventions tool. Risk of bias of seven categories (C=confounding, P=participant selection bias, I=intervention bias, D=deviation from intervention, MD=missing data, OM=outcome measurement bias, OR=outcome reporting bias). Results are colour coded and highlighted according to scored risk of bias; low (L) risk of bias is represented in green, moderate (M) risk of bias is represented in yellow, and serious (S) risk of bias is represented in orange.

|  | **C** | **P** | **I** | **DI** | **MD** | **OM** | **OR** |
| --- | --- | --- | --- | --- | --- | --- | --- |
| Amano et al, 2016 | L | L | L | L | M | L | M |
| van der Heijden et al., 2016 | L | L | L | L | S | L | L |
| Apprich et al., 2012 | L | M | L | L | L | M | L |
| Apprich et al 2010 | L | L | L | L | M | M | L |
| Bae et al, 2015 | L | L | L | L | L | M | M |
| Baum et al., 2012 | L | L | L | L | L | M | M |
| Baum et al, 2013 | L | L | L | L | M | M | M |
| Baum et al., 2012 | L | L | L | L | L | M | M |
| Mostrom et al., 2014 | S | L | L | L | L | M | L |
| Bining et al., 2009 | L | S | M | L | S | S | L |
| Bolbos et al., 2008 | L | L | L | L | L | S | L |
| Farrokhi et al., 2011 | L | L | L | L | L | M | L |
| Gheno et al., 2016 | L | M | M | L | L | M | M |
| van Ginckel et al., 2013 | L | L | L | L | L | L | L |
| Gupta et al., 2014 | L | L | L | L | L | M | M |
| Haughom et al., 2012 | M | L | L | L | L | M | L |
| Hovis et al., 2011 | L | L | L | L | L | M | M |
| Joseph et al., 2011 | L | L | L | L | L | M | M |
| Kai et al., 2011 | S | S | L | L | M | L | M |
| Kang et al., 2016 | M | L | L | L | L | L | L |
| Lansdown et al., 2015 | S | L | L | L | L | M | L |
| Lau et al., 2016 | L | L | L | L | L | M | L |
| Liebl et al., 2014 | M | M | M | L | L | M | M |
| Li et al., 2013 | M | L | L | L | L | M | L |
| Li et al., 2011 | L | L | L | L | L | M | L |
| Matsubara et al., 2015 | S | S | M | L | L | S | L |
| Mosher et al., 2004 | L | L | L | L | L | L | L |
| Okazaki et al., 2015 | L | L | L | L | L | L | L |
| Osaki et al., 2015 | M | L | L | L | L | L | M |
| Palmieri-Smith et al., 2016 | L | L | L | L | L | M | L |
| Pedoia et al., 2016 | L | L | L | L | L | M | M |
| Pedoia et al., 2016 | L | L | L | L | L | M | M |
| Pedoia et al., 2017 | L | L | L | L | L | M | M |
| Rehnitz et al., 2014 | M | S | M | L | S | M | L |
| Russell et al., 2017 | M | M | L | L | S | M | M |
| Sauerschnig et al., 2014 | L | L | L | L | L | M | L |

Appendix 2, continued

| Snoj et al., 2016 | M | S | L | L | L | M | M |
| --- | --- | --- | --- | --- | --- | --- | --- |
| Subhawong et al., 2014 | L | M | M | L | M | M | L |
| Su et al., 2013 | L | L | L | L | L | M | M |
| Su et al., 2016 | M | L | L | L | S | M | M |
| Theologis et al., 2011 | M | L | L | L | L | M | M |
| Thuiller et al., 2013 | L | L | L | L | L | M | L |
| Wirth et al., 2016 | L | M | L | L | L | L | M |
| Witschey et al., 2010 | S | M | M | S | L | S | L |
| Xu et al., 2011 | L | M | M | M | M | M | L |
| Zaid et al., 2015 | L | L | L | M | L | M | L |
| Kim et al., 2018 | L | L | L | L | L | M | L |
| Kogan et al., 2018 | L | L | L | L | L | M | L |
| Mostrom et al., 2017 | L | L | L | L | L | M | L |
| Pfeiffer et al., 2017 | S | L | L | L | L | M | L |
| Pietrosimone et al., 2017 | L | L | L | L | M | M | L |
| Tao et al., 2018 | L | L | L | L | L | M | L |
| Teng et al., 2017 | L | L | L | L | S | M | L |
| Wang et al., 2018 | L | L | L | L | S | M | M |
| Collins et al., 2018 | L | L | L | L | L | M | L |

Appendix 3. Table listing sensitivity analyses excluding studies using Osteoarthritis Initiative (OAI) data and within-groups study designs for each respective compartment. Effect size (ES) indicates the standardized mean difference (SMD) of the excluded studies, ES of the primary analysis represents the SMD of the remaining studies not excluded.

OAI=Osteoarthritis Initiative, MF=medial femur, MT=medial tibia, LF=lateral femur, LT=lateral tibia, P=patella, TrF=trochlea, ES=effect size, SMD=standardized mean difference.

| **Analysis** | **Outcome** | **Articles** | **Compartment** | **ES (SMD)** | **ES Primary Analysis (SMD)** |
| --- | --- | --- | --- | --- | --- |
| OAI | T2 | 7 | MF | 0.51 [0.36–0.67]; p=0.001 | 0.69 [0.31–1.07]; p=0.001 |
|  |  | 5 | MT | 0.54 [0.16-0.92]; p=0.005 | 0.60 [0.13-1.06]; p=0.01 |
|  |  | 3 | LF | 0.41 [0.18–0.64]; p=0.001 | 0.78 [0.27–1.29]; p=0.003 |
|  |  | 3 | LT | 0.74 [0.17–1.30]; p=0.01 | 0.82 [0.36–1.30]; p=0.001 |
|  |  | 3 | P | 0.05 [-0.39–0.49]; 0.8 | 0.51 [0.14–0.88]; p=0.008 |
| Within-groups | T2 | 2 | MF | 0.24 [-0.28–0.76]; p=0.37 | 0.66 [0.39–0.93]; p=0.001 |
|  |  | 2 | MT | 0.09 [-0.25–0.44]; p=0.6 | 0.63 [0.26–1.0]; p=0.001 |
|  |  | 2 | LF | 0.13 [-0.22–0.47]; p=0.48 | 0.78 [0.34–1.21]; p=0.001 |
|  |  | 2 | LT | 0.31 [-0.04–0.66]; p=0.08 | 0.88 [0.47–1.29]; p=0.001 |
|  |  | 2 | P | -0.002 [-0.33–0.33]; p=0.99 | 0.47 [0.13–0.81]; p=0.006 |
|  |  | 1 | TrF | 0.67 [0.28–1.05]; p=0.001 | 0.84 [0.33–1.35]; p=0.001 |
|  | T1ρ | 4 | MF | 0.25 [-0.06–.56]; p=0.1 | 0.44[0.17–0.71]; p=0.001 |
|  |  | 4 | LF | 0.28 [0.007–0.54]; p=0.04 | 0.44[0.21–0.67]; p=0.001 |
|  |  | 4 | MT | -0.13 [-0.46–0.20]; p=0.43 | 0.29[0.04–0.54]; p=0.02 |
|  |  | 3 | LT | -0.46 [-1.78–0.86]; p=0.5 | 0.14[-0.15–0.42]; p=0.34 |
|  |  | 3 | P | -0.12 [-0.43–0.18]; p=0.4 | 0.13[-0.11–0.38]; p=0.29 |
|  |  | 1 | TrF | 0.19 [-0.19–0.56]; p=0.3 | .13[-0.28–0.550; p=0.5 |

Appendix 4. Table listing subgroups analyses excluding studies analyzing effect sizes of groups with specific risk factors for each respective compartment, including patients with anterior cruciate ligament (ACL)injury, patients with risk factors for patellofemoral osteoarthritis (OA), and patients with articular cartilage injuries. Effect size (ES) indicates the standardized mean difference (SMD) of the excluded studies, ES of the primary analysis represents the SMD of the remaining studies not excluded.

ACL= anterior cruciate ligament, OA=osteoarthritis, ES=effect size, SMD=standardized mean difference, MF=medial femur, MT=medial tibia, LF=lateral femur, LT=lateral tibia, P=patella, TrF=trochlea, ES=effect size, SMD=standardized mean difference.

| **Subgroup** | **Outcome** | **Articles** | **Compartment** | **ES (SMD)** | **ES Primary Analysis (SMD)** |
| --- | --- | --- | --- | --- | --- |
| ACL Injury | T2 | 8 | MF | 0.66 [0.11–1.21]; p=0.02 | 0.62 [0.34–0.91]; p=0.001 |
|  |  | 9 | MT | 0.34 [-0.05–0.73]; p=0.08 | 0.79 [0.29–1.30]; p=0.002 |
|  |  | 7 | LF | 0.37 [-0.11–0.85]; p=0.1 | 0.91 [0.36–1.47]; p=0.001 |
|  |  | 7 | LT | 0.35 [0.09–0.62]; p=0.009 | 1.16 [0.61–1.71]; p=0.001 |
|  |  | 3 | P | 0.18 [-0.12–0.47]; p=0.24 | 0.45 [0.08–0.81]; p=0.02 |
|  |  | 2 | TrF | 0.74 [0.41–1.07]; p=0.001 | 0.67 [-0.16–1.49]; p=0.11 |
|  | T1ρ | 10 | MF | 0.34 [0.14–0.53]; p=0.001 | 0.56 [-0.18–1.30]; p=0.14 |
|  |  | 9 | LF | 0.38 [0.18–0.57]; p=0.001 | 0.82 [-0.54–2.17]; p=0.2 |
|  |  | 11 | MT | 0.05 [-0.16–0.26]; p=0.7 | 0.73 [0.17–1.29]; p=0.01 |
|  |  | 8 | LT | -0.14 [-0.71–0.43]; p=0.6 | 1.37 [0.11–2.62]; p=0.03 |
|  |  | 6 | P | -0.11 [-0.35–0.12]; p=0.35 | 0.28 [-0.02–0.58]; p=0.06 |
|  |  | 3 | TrF | 0.19 [-0.10–0.48]; p=0.2 | -0.13 [-1.15–0.88]; p=0.8 |
| Patellofemoral OA Risk | T2 | 2 | MF | 0.18 [-0.36–0.71]; p=0.52 | 0.68 [0.41-0.95]; p=0.001 |
|  |  | 2 | LF | 0.06 [-0.35–0.47]; p=0.8 | 0.79 [0.35–1.22]; p=0.001 |
|  |  | 7 | P | 0.29 [0.06–0.51]; p=0.01 | 0.53 [0.03–1.02]; p=0.04 |
|  | T1ρ | 1 | MF | 0.02 [-0.32–0.36]; p=0.9 | 0.42 [0.22–0.63]; p=0.001 |
|  |  | 1 | LF | 0.26 [-0.09–0.60]; p=0.1 | 0.40 [0.21–0.60]; p=0.001 |
|  |  | 3 | P | 0.28 [-0.02–0.58]; p=0.06 | -0.11 [-0.35–0.12]; p=0.3 |
|  |  | 1 | TrF | -0.13 [-1.15–0.88]; p=0.8 | 0.19 [-0.1–0.48]; p=0.21 |
| Articular Cartilage Injury | T2 | 3 | MF | 1.46 [-0.3–3.23]; p=0.1 | 0.49 [0.30–0.68]; p=0.001 |
|  |  | 2 | LF | 3.21 [2.24–4.18]; p=0.001 | 0.39 [0.14–0.64]; p=0.003 |
|  |  | 2 | MT | 1.29 [-1.59–4.17]; p=0.38 | 0.50 [0.21–0.79]; p=0.001 |
|  |  | 2 | LT | 2.88 [2.32–3.44]; p=0.001 | 0.55 [0.31–0.78]; p=0.001 |
|  |  | 2 | P | 1.80 [-0.71–4.31]; p=0.16 | 0.23 [0.06–0.39]; p=0.007 |

Appendix 5. Preferred Reporting in Systematic Reviews and Meta-Analysis 2009 Checklist.

| **Section/topic** | **#** | **Checklist item** | **Reported on page #** |
| --- | --- | --- | --- |
| **TITLE** | | |  |
| Title | 1 | Identify the report as a systematic review, meta-analysis, or both. | Title |
| **ABSTRACT** | | |  |
| Structured summary | 2 | Provide a structured summary including, as applicable: background; objectives; data sources; study eligibility criteria, participants, and interventions; study appraisal and synthesis methods; results; limitations; conclusions and implications of key findings; systematic review registration number. | Abstract |
| **INTRODUCTION** | | |  |
| Rationale | 3 | Describe the rationale for the review in the context of what is already known. | 4-5 |
| Objectives | 4 | Provide an explicit statement of questions being addressed with reference to participants, interventions, comparisons, outcomes, and study design (PICOS). | 5 |
| **METHODS** | | |  |
| Protocol and registration | 5 | Indicate if a review protocol exists, if and where it can be accessed (e.g., Web address), and, if available, provide registration information including registration number. | 5 |
| Eligibility criteria | 6 | Specify study characteristics (e.g., PICOS, length of follow-up) and report characteristics (e.g., years considered, language, publication status) used as criteria for eligibility, giving rationale. | 6 |
| Information sources | 7 | Describe all information sources (e.g., databases with dates of coverage, contact with study authors to identify additional studies) in the search and date last searched. | 6 |
| Search | 8 | Present full electronic search strategy for at least one database, including any limits used, such that it could be repeated. | Appendix 1 |
| Study selection | 9 | State the process for selecting studies (i.e., screening, eligibility, included in systematic review, and, if applicable, included in the meta-analysis). | 6-7 |
| Data collection process | 10 | Describe method of data extraction from reports (e.g., piloted forms, independently, in duplicate) and any processes for obtaining and confirming data from investigators. | 7 |
| Data items | 11 | List and define all variables for which data were sought (e.g., PICOS, funding sources) and any assumptions and simplifications made. | 7 |
| Risk of bias in individual studies | 12 | Describe methods used for assessing risk of bias of individual studies (including specification of whether this was done at the study or outcome level), and how this information is to be used in any data synthesis. | 7-8 |

| Appendix 5, continued | | | | | |
| --- | --- | --- | --- | --- | --- |
| Summary measures | 13 | | State the principal summary measures (e.g., risk ratio, difference in means). | | 8 |
| Synthesis of results | 14 | | Describe the methods of handling data and combining results of studies, if done, including measures of consistency (e.g., I^2^) for each meta-analysis. | | 8-9 |
| Risk of bias across studies | | 15 | | Specify any assessment of risk of bias that may affect the cumulative evidence (e.g., publication bias, selective reporting within studies). | 8 |
| Additional analyses | | 16 | | Describe methods of additional analyses (e.g., sensitivity or subgroup analyses, meta-regression), if done, indicating which were pre-specified. | 8-9 |
| **RESULTS** | | | | |  |
| Study selection | | 17 | | Give numbers of studies screened, assessed for eligibility, and included in the review, with reasons for exclusions at each stage, ideally with a flow diagram. | 9-10, Figure 1 |
| Study characteristics | | 18 | | For each study, present characteristics for which data were extracted (e.g., study size, PICOS, follow-up period) and provide the citations. | 10, Table 1 |
| Risk of bias within studies | | 19 | | Present data on risk of bias of each study and, if available, any outcome level assessment (see item 12). | 10 |
| Results of individual studies | | 20 | | For all outcomes considered (benefits or harms), present, for each study: (a) simple summary data for each intervention group (b) effect estimates and confidence intervals, ideally with a forest plot. | Figures 2-7 |
| Synthesis of results | | 21 | | Present results of each meta-analysis done, including confidence intervals and measures of consistency. | Figures 2-7 |
| Risk of bias across studies | | 22 | | Present results of any assessment of risk of bias across studies (see Item 15). | 10, Appendix 2 |
| Additional analysis | | 23 | | Give results of additional analyses, if done (e.g., sensitivity or subgroup analyses, meta-regression [see Item 16]). | 12-15 |
| **DISCUSSION** | | | | |  |
| Summary of evidence | | 24 | | Summarize the main findings including the strength of evidence for each main outcome; consider their relevance to key groups (e.g., healthcare providers, users, and policy makers). | 15-17 |
| Limitations | | 25 | | Discuss limitations at study and outcome level (e.g., risk of bias), and at review-level (e.g., incomplete retrieval of identified research, reporting bias). | 15-16 |
| Conclusions | | 26 | | Provide a general interpretation of the results in the context of other evidence, and implications for future research. | 19 |
| **FUNDING** | | | | |  |
| Funding | | 27 | | Describe sources of funding for the systematic review and other support (e.g., supply of data); role of funders for the systematic review. | 20 |
